# A systematic approach to classifying and evaluating heterogeneity measures

**DOI:** 10.1098/rsos.242047

**Published:** 2025-10-22

**Authors:** Ramona Ottow

**Affiliations:** ^1^ Pompeu Fabra University, Barcelona, Spain

**Keywords:** centralization, exponential network, graph index, inequality, irregularity, power-law network

## Abstract

This study introduces a systematic framework for analysing heterogeneity through three principal measure classes: dispersion-based, expected-difference and divergent approaches. I demonstrate that these classes capture distinct structural aspects, with graph heterogeneity measures incorporating global topology beyond degree counts while degree-focused approaches quantify connectivity variation. Key findings establish that apparent inconsistencies across measures reflect heterogeneity’s complex nature rather than methodological flaws. The framework enables context-appropriate measure selection for applications ranging from epidemiological modelling to cyber security, while highlighting the critical distinction between degree-focused and topology-aware heterogeneity quantification. The work advances network science by mapping methodological trade-offs and proposing future development of tunable hybrid measures for complex systems analysis.

## 1. Introduction: heterogeneity in networks

Heterogeneity is a key concept in network science, but there is a lack of clarity and consensus in the literature as to how to define and quantify it. An important difference is the distinction between graph heterogeneity, which looks at the overall structural differences and irregularities in the whole network, and degree heterogeneity, which specifically examines the variability in the number of connections each node has. Based on many existing measures, the level of heterogeneity affects the dynamics of disease spread on social contact networks, the robustness to attacks on computer networks and the stability of ecological systems [1–3]. The scientific areas in which the concept is used are as diverse as the definitions and approaches to measure it. Networks with degrees that follow a Poisson distribution are often characterized as homogeneous, and ones that follow a power-law (PL) distribution as heterogeneous. However, depending on the chosen heterogeneity measure, there exist cases in which networks of each kind have the same heterogeneity value [4]. So far, there have been efforts to compare commonly used measures and derive bounds [5–8], but a comprehensive up-to-date overview, categorization and evaluation of degree and graph heterogeneity definitions, measures and demands is missing. Filling this gap is important to provide guidance and consistency to researchers when choosing the appropriate heterogeneity measure for their work and thus improving predictions in epidemiology, computer security, ecology and many other fields.

In this work, I start by collecting common definitions of graph and degree heterogeneity and show that they can be grouped into three main categories: (1) degree heterogeneity; (2) topological graph heterogeneity; and (3) centrality and position-based graph heterogeneity.

I further provide an overview of the diverse criteria and requirements that researchers place on the measures. These can be grouped into: (i) general properties, like aiming for a measure that is a single, unique value; (ii) local topological properties, focusing on incorporation of local information, like differences in neighbouring degrees; (iii) global properties, which deal mainly with claims that the measures should recognize specific types of networks as hetero- or homogeneous; and (iv) properties related to the behaviour of the measure to manipulation of the network, like rewiring of edges.

I classify the existing heterogeneity quantitative measures conceptually into three classes. The first class comprises dispersion-based approaches, which mainly consider heterogeneity as the deviation from regularity or variation within the set of degrees, and aggregate degree differences. The second comprises expected-difference-based approaches, which are inspired by the Gini coefficient and use the expected absolute difference in degrees as the underlying concept to measure heterogeneity. The third class comprises divergent approaches, which take a completely different approach.

Finally, I systematically evaluate and discuss each class accordingly to the desired properties. I provide analytical proofs where possible, with general conditions outlined otherwise. In cases, where measures do not fulfil properties, I present counter-examples for specific measures. Additionally, I use simulations to explore properties related to random graph models, also for specific measures. This discussion and explanation of reasons behind breaches or compliance with requirements can enhance the understanding of measures and their real-world applicability.

### 1.1. Challenges in measurement development

Four main challenges can be identified in the literature regarding heterogeneity in networks. The most fundamental is the definition of heterogeneity itself. At the moment there exist many different understandings of homogeneity and heterogeneity, not only between different fields of study, but also within the same [4,9,10]. Some definitions have a node-level perspective, focusing on degree variability. Others take a network-level perspective, focusing on the shape of the degree distribution, structural properties like connection patterns, regularity or topology of the network. These different ways of understanding heterogeneity can lead to errors and difficulties in the development and application of heterogeneity measures. For instance, if it is understood that heterogeneity is not only based on node degrees, but should also consider the associated topological structure, selecting a measure that accounts exclusively for degrees may lead to inaccuracies. Certain measures yield identical values for networks with identical degree distributions but different topologies. Consequently, this mismatch can lead to incorrect predictions about how information or diseases spread, since these processes often depend on more than just the number of connections [11]. It is important to distinguish between *degree* heterogeneity, a concept that focuses on the degree variability, and *graph* heterogeneity, which focuses on more broader structural features.

The second problem concerns translation from definition to quantification. The measure needs to actually capture what is intended, while ensuring that measures align with the definition. For example, if a graph is defined as heterogeneous based on the existence of nodes with significantly larger degree than the average degree of the network, the developed measure must capture this property. The definition should be correctly translated to the formula for the measure.

The third problem is the kind of quantification of heterogeneity. There are different approaches that call their developed formula an index, indicator, measure, metric or score [4,9,10,12]. The clarification and verification of the definition is often missing, so that it is not clear if, for example, a developed ‘measure’ is indeed a measure in the mathematical (non-negativity, σ-additivity, etc.) or colloquial sense. For example, a statistical index is a statistic that combines several indicators or variables to a single values, like the Human Development Index, with the aim of simplifying and summarizing complex issues. On the other hand, mathematical metrics are used to quantify distances or dissimilarities between elements, for example distances between places in route planning.

The fourth challenge concerns the desirable properties. It is not clear what desired or necessary properties a measure should actually have. In a few works on heterogeneity measures are the minimum value set to zero and defined as homogeneity, but the maximal value is unknown or missing. For example, in [13] the measure yields zero for regular graphs, which are assumed to be homogeneous. All networks with non-zero values are heterogeneous. On the one hand this dichotomous assignment is very simple but on the other hand, it becomes impossible to rank networks according to how heterogeneous they are. Exceptional contributions to this area include the work of [14] and [6], which provide valuable properties by examining measures for various graph classes and determining their extreme values, while [7] makes a significant contribution by developing fundamental principles that heterogeneity measures should ideally satisfy.

### 1.2. Notation

In this section, I introduce the basic symbols and notation that I will use throughout the rest of the article.

Given a finite undirected graph (or network) G=(V,E) with no loops and multiple edges and |V|=n<∞ nodes and $|E|=m\leq \frac{1}{2}n(n-1)$ edges. dG=m(n2)=2mn(n−1) is called the density of G and ${ℵ}_{1}$ is the largest (or dominant) eigenvalue of the graph’s adjacency matrix.

The number of incident edges of a node i is called its degree $k_{i}$. The mean degree of the graph is denoted as $\bar{k}=n^{-1}\sum_{i}k_{i}$ and the set of all node degrees by $K=\left\{k_{1},\ldots ,k_{n}\right\}$. The degrees are distributed according to the degree distribution $P(k)=\frac{n_{k}}{n}$, where $n_{k}$ is the number of nodes with degree k.

A graph G is called an r-regular graph if all nodes have the same degree r and a star graph consists of one central node and all other nodes of the network are only connected to that central node. Diverse graphs, on the other hand, are characterized by a broad node degree distribution, indicating that the nodes have a wide range of degrees rather than a single dominant hub [10]. A completely diverse graph is defined as a diverse graph with only one non-unique degree value.

In this paper, I refer to any formula that aims to quantify graph or degree heterogeneity as ‘measure’ without strict compliance to the mathematical definition of a measure in measure theory.

## 2. Definitions of heterogeneity

In the literature, heterogeneity is described using a wide range of definitions, which I group into two distinct, but related concepts: degree heterogeneity and graph heterogeneity.

Degree heterogeneity is commonly conceptualized as the dispersion or diversity in degrees, capturing the range and variety of degrees of a network (presented in §2.1).

Graph heterogeneity, on the other hand, is conceptualized from two main perspectives: first, a topological-based approach that focuses on irregularities not only in degrees, but also includes local information on degree disparities (presented in §2.2); and second, a centrality and position-based approach that emphasizes the position of nodes within a network (presented in §2.3).

The following sections present the different definitions found in the literature.

### 2.1. Degree heterogeneity

This set of definitions characterizes heterogeneity by degree variation and is grounded in three concepts: inequality, dispersion/diversity and irregularity.

Hu & Wang [4] define heterogeneity as inequality in node degrees, corresponding to the idea of income inequality in economics. It is commonly quantified using the Lorenz curve and Gini coefficient, which are classical tools for assessing distributional inequality [15]. This aligns with [7], who emphasize that heterogeneity corresponds to distributional inequality, requiring heterogeneity measures to consistently reflect changes in the equality/inequality of a distribution.

A second approach to define degree heterogeneity focuses on spread and diversity in connectivity. Snijders [16] defines degree heterogeneity through dispersion of the degrees, measured by the degree variance, where greater dispersion in node degrees corresponds to higher heterogeneity.

Liu *et al*. [17] emphasize the disparity between low- and high-degree nodes in network controllability. They hypothesize that high degree nodes have an important role in many processes on networks, such as the stability of networks, the diffusion on networks, etc., and controlling these therefore must be a crucial part in controlling the network. Liu *et al*. [17] are interested in the relationship between the cardinality of the set of driver nodes, which are those nodes that can direct network dynamics, and high degree nodes and they find that driver nodes typically are not high degree nodes. They define heterogeneity as the disparity in connectivity between these groups.

A distinct angle is introduced by [10], who differentiate between dispersion and *diversity* of node degrees. As an example, one can consider the degrees of a star network to have high dispersion because the degree range is high, but since only two different degrees exist, the diversity in degrees is low.

A third perspective, rooted in graph theory, defines degree heterogeneity through the concept of *Abweichung von der Regularität* (German for ‘deviation from regularity’). Von Collatz & Sinogowitz [13] derive bounds for the largest eigenvalue for finite, connected graphs. They find that the mean degree is smaller or equal to the largest eigenvalue while equality holds if and only if the graph is regular. They propose the difference between these values as a measure of irregularity, called *Defekt* (German for ‘defect’), which clearly has a lower bound of zero for regular graphs. Many following research adopt this view considering irregularity, i.e. a deviation from regularity as one aspect of heterogeneity [6,7,18].

### 2.2. Topological graph heterogeneity

While §2.1 examines heterogeneity through node degree distributions, the following definition focuses additionally on structural irregularities arising from topological relationships, emphasizing how edges and node neighbourhoods collectively shape structural irregularities.

Estrada [18] defines graph heterogeneity as deviations arising from both the degree distribution (e.g. hubs in scale-free networks), and local edge configurations (e.g. hubs linked to low-degree versus high-degree nodes). This dual perspective captures a ‘higher-order’ heterogeneity: two networks with identical degree distributions can exhibit distinct graph heterogeneity levels depending on edge arrangements.

### 2.3. Centrality and position-based graph heterogeneity

The second group of graph heterogeneity definitions is based on the idea of centrality and node positions. Bavelas [19] works on the problem of communication in team-work, in particular on the communication pattern and the effect on the group’s performance. They define the distance between group members as the minimum number of other members in the communication path between both, plus one. They assume that this distance as well as the communication structure between all group members, with respect to distance, is related to the communication performance between them and within the group. The closer, that is the smaller the number of other members in the communication path, a person is to one or more colleagues, the better the communication and the more central is this person. The more distant all workers are from each other, the more complex and heterogeneous is the network. Therefore, Bavelas [19] defines heterogeneity based on node positions.

Although the author’s work is not about structural or degree variations in a network, I include it in this work, because it is the first definition on which many centralization measures are based. One example of these subsequent measures is the work of [14]. They approach the topic from the perspective of social science theory and formalize their ideas of social structure on integration, polarization and centralization through graphs and graph theory. First, they define node centrality based on position in the network (central or peripheral) and define graph heterogeneity as the disparity between central and peripheral nodes (centralization). A graph is called heterogeneous (centralized) if there are great differences in the node positions.

The next definition in this line of work is the commonly used Freeman centrality measure from 1978 [20]. Building upon the foundational work of [19] and [14], [20] similarly defines heterogeneity based on the position or centrality of nodes in relation to the most central node. They define heterogeneity as the centralization of a network—the extent to which the most central node tends to be more central than all other nodes in the network. This approach aligns with numerous other studies in social network communication [21–25].

The next definition in this line of work is the commonly used Freeman centrality measure from 1978 [20]. Like [19] and [14], as well as many others coming from communication in social networks [20–25] define heterogeneity based on the position or centrality of nodes in relation to the most central node. They define heterogeneity as the centralization of a network—the extent to which the most central node tends to be more central than all other nodes in the network.

Finally, [12] follows the centrality-based definitions of [20], but their work leads to a new perspective. Butts [12] extends Freeman’s work and finds that heterogeneity in a network, considering the network’s density and size, is reflected by the excess maximum degree over and above its minimal possible value in relation to the maximal possible excess.

Centrality and position-based definitions concern graph heterogeneity and focus on structural properties in relation to the node’s positions within the network and aim to capture properties, like diversity in communication paths or node roles.

## 3. Desirable and necessary measure properties

This section provides an overview of the properties that researchers deem desirable or necessary in formulating their proposed heterogeneity measures. This includes properties of obtaining extreme measurement values for specific graphs, but I do not include research on finding their bounds here. I also omit discussion of properties that are based on specific applications, as it is beyond the scope of this article.

Section 3.1 describes general properties, §3.2 describes local properties, mainly those focusing on relationships between degrees, §3.3 describes global properties, which mainly connect demands to specific graphs or random graph models, and §3.4 describes properties of how heterogeneity measures should behave in case of changes in the input network.

### 3.1. General properties

A graph heterogeneity measure should quantify the graph’s heterogeneity [18], be a single, unique parameter or value [18,26] and be interpretable [12,20]. The measure should furthermore allow the comparison between a variety of networks and be applicable to different network types [7]. In particular, the requirement of independence of degree distribution is widely demanded for both graph [4,26] and degree heterogeneity measures [10]. The measure should enable the comparison of networks with entirely different degree distributions and be applicable to networks that can fit various degree distribution models [18].

### 3.2. Local topology properties

Local topological properties on heterogeneity measures focus on the relationship between node degrees rather than the global topology of the network. The most common criteria concerning these relationships can be organized into two distinct groups. The first group requires that a graph heterogeneity measure should account for the extent to which the highest degree or a group of high-degree nodes exceeds all others [12,20]. The second group demands that a heterogeneity measure should capture differences in degrees between nodes—whether local or global, weighted or relative [16,18,20].

### 3.3. Global properties

Global properties concern the behaviour of measures for specific graphs or topologies. In terms of graph heterogeneity, the measure should increase from lowest values for regular graphs (considered as homogeneous) to Erdős–Rényi (ER) networks to scale-free networks (PL) with decreasing degree exponent (considered as heterogeneous) [17,18,26]. This corresponds to the claims of [18,26] that regular graphs are homogeneous while star-like graphs are heterogeneous.

By contrast, when considering degree heterogeneity, a different approach is proposed by [10]. They argue that a degree heterogeneity measure should reach its maximum for networks where all nodes except one have distinct degrees, with only two nodes sharing the same degree. This alternative view emphasizes capturing the diversity of node degrees, focusing on the variety of different degrees present in a network.

### 3.4. Behaviour to manipulation

The final group of properties stated by researchers considers the response of measures to small changes in the network. Badham [7] developed three principles that they require. The transfer principle states that the heterogeneity measure should decrease when an edge from a given node is rewired from a higher-degree node to another node with a lower degree, without reversing their degree order. This principle reflects the idea that such a change reduces the disparity in node degrees within the network, thereby lowering its overall heterogeneity. The addition principle considers the situation in which all node degrees are increased by the same amount. It claims that either the heterogeneity measure does not change, which would be the case for measures based on variation about the mean or absolute differences, or that the measure decreases, which is the case for relative measures [7]. The third principle is called the replication principle. Badham [7] claims for global and local measures to be the same for replications of the network (integer multiple or a non-inter multiple), independent of the number of replications. For instance, for a given network with a specific degree distribution, one could create a second network that consists of two components, where each component is the initial network. This new network has twice the amount of each degree of the initial network, but the degree distribution is the same. The principle claims that for the initial and for the replicated (here doubled) network, the measures should be the same. In case of non-integer replication, the problem arises that the number of node degrees in the new network might be non-integer. This is solved by scaling all counts with the smallest value that ensures an integer count.

## 4. Classification of existing measures

In this section, I introduce three main classes of heterogeneity measures. This classification is based on the general mathematical formulas that group measures according to their construction methods, rather than the definitions from §2.

The first class, dispersion aggregation (§4.1), includes three variants: global (deviations from a network statistic), adjacent pairwise (differences between neighbouring nodes), and pairwise (differences between all node pairs). The second class, expected difference (§4.2), comprises measures based on the expected degree differences, inspired by the Gini coefficient, with adjacent pairwise and pairwise variants. The third class, special approaches (§4.3), covers unique formulations not included in the previous classes.

Table 1 summarizes how these classes relate to the definitions introduced earlier. The following subsections provide a detailed presentation of each class.

**Table 1.** Mapping of heterogeneity measure classes to heterogeneity definitions (§2).

class	definitions (§2)	section
**dispersion aggregation**		§4.1
global	centrality/position-based graph heterogeneity (§2.3) and degree heterogeneity (§2.1)	
adjacent pairwise	topological graph heterogeneity (§2.2)	
pairwise	degree heterogeneity (§2.1)	
**expected difference**		§4.2
adjacent expected	topological graph heterogeneity (§2.2)	
pairwise expected	degree heterogeneity (§2.1)	
**special approaches**	topological graph heterogeneity (§2.2) and degree heterogeneity (§2.1)	§4.3

### 4.1. Dispersion aggregation

This section contains three subclasses. The first class in §4.1.1 focuses on measures that concerns degree heterogeneity as a deviation from or in relation to a network statistic. The second and third classes (§4.1.2 and §4.1.3) address measures by aggregating local differences in node degrees, either by adjacent (graph heterogeneity) or across all pairs of nodes (degree heterogeneity).

#### 4.1.1. Global dispersion aggregation

Measures in this class consider degree heterogeneity as the deviation of all degrees from a specific network statistic of interest, like the maximum or mean degree. These measures can be described by the general formula:


(4.1) $$\begin{array}{lcr} & c\sum_{i=1}^{n}g(k_{i}-h(K)), & \end{array}$$

where c is a scaling factor, for example the inverse of the number of nodes, $g:\mathbb{R}\;\overset{\;}{\to }\;{\mathbb{R}}_{\geq 0}$ a convex function with global minimum at zero, usually square or absolute value, and $h:K\;\overset{\;}{\to }\;{\mathbb{R}}_{\geq 0}$ a constant function of the node degrees, representing the statistic of interest, like the maximum or average. Employing a convex function with minimum value obtained at zero gives less weight to differences that are small and higher weight to larger differences.

##### 4.1.1.1. Statistic of interest: maximum degree

Approaches falling under this group are among the first that researchers developed to measure graph heterogeneity in networks. Reference [19] and in particular [14] introduce the concept of centrality and centralization (compare definitions in §2.3, which attracted a lot of attention and advancement over following years [27–29]). The idea is that each node has a specific centrality property that measures its position within the network ranging from ‘not central’, peripheral to most central, or important. Centralization then refers to their dispersion throughout the global network topology in relation to the most central node. Centralization is an aggregate of the local centralities that measures to what extent node centralities differ from the most central node. Network topologies that show a large value and therefore are considered as centralized are said to be heterogeneous and those with a smaller value homogeneous.

Bavelas [19] defines the centrality of a node as the sum of all distances from that node to all others. Their work initiated the development of many other ways to define the centrality of a node in a network and therefore as well the ways to define centralization of a whole network [14,20].

One of these is the work of [14]. Like [19] they start with a distance-based view on centrality, but adapt it to a degree-based, which means that the centrality of a node is defined by the node degree. As a result, the developed measure captures degree heterogeneity rather than overall graph heterogeneity. The measure quantifies how dispersed the node positions (whether peripheral or central) are, relative to the most central node in the graph.

They define the degree of a node as measure of local centrality $k_{i}$ (degree) of the node i and the degree-based centralization measure as the sum over all differences in node centrality to the most central node:


(4.2)HHG(G):=∑i=1n(−1)(ki−kmax),(4.3)=∑i=1n(kmax−ki).

Note that this expression appears in the general formula (4.1) having a scaling parameter c≡1, negative identity function g and the maximum function h.

The smaller this sum, the more similar the nodes in the network are to the most central node, and the more homogeneous the degrees become. Conversely, the larger the sum, the more nodes differ in centrality from the most central node, meaning that a higher dispersion in node centralities is interpreted as indicating higher heterogeneity in degrees.

Freeman [20] derived their measure from a local view of centrality to a global one. They start deriving three possible local centrality measures from the star graph—since, in their view, the star graph represents an extreme example of a heterogeneous network—and then derive requirements on their graph heterogeneity measure. The middle node in a star graph is unique and special (most central) because of three reasons. First, it has the highest attainable degree (degree-centrality). Second, it lies along the shortest route connecting the greatest number of node pairs (betweenness-centrality). Third, it is close with respect to distance to all other nodes (closeness-centrality). For each of these three local centralities, [20] create a global measure. They require that the global measure both (i) quantifies how much more important the most central node is compared with all other nodes, and (ii) is expressed as a ratio relative to its maximum possible value so that it attains its extreme values in the most extreme cases. For the degree centrality the degree heterogeneity measure by [20] is:


(4.4)HF(G):=1max∑i=1n(kmax−ki)∑i=1n(kmax−ki),(4.5)=1max∑i=1n(kmax−ki)HHG(G).

The measure is therefore a scaled version of the Høivik & Gleditsch [14] measure with scaling factor $c^{-1}=\max\;\sum_{i}^{n}(k_{\max}-k_{i})$, which is the smallest total difference for a graph with n points, no matter how many connections it has. They concluded that the maximum sum in the denominator is reached for a star or wheel graph with value $(n-2)(n-1)=n^{2}-3n+2$. Furthermore, in case of a five star node [20] prove that the measure is maximal for star or wheel graphs, minimal for circle or complete graphs, but also show that between these extremes are remarkable differences.

The most recent approach by [12] renormalizes the Freeman [20] centrality and achieves a relative measure for fixed density and graph size that measures the excess maximum degree over the minimal possible maximum degree. The reason is that there is only a feasible area for centralization scores, given a density and size of a network, for example owing to combinatorial reasons. Therefore it is necessary, that if one normalizes the measure, one should take the bounds of that area into account, so that the measure is better interpretable and useful. Butts [12] reformulated the Freeman [20] formula, depending on maximum degree and density and uses an affine transformation for normalizing it. They derive and employ bounds on the maximum degree, depending on the density, to create a density-normalized centralization score from the reformulated score:


(4.6)HB(G):=HHG(G)−(min{G′:|V|=n,dG′=dG}HHG(G′))(max{G′:|V|=n,dG′=dG}HHG(G′))−(min{G′:|V|=n,dG′=dG}HHG(G′)),(4.7)=kmax−⌈dG(n−1)⌉(n−1)(dG+min[1−dG,dG(n2−1)])−⌈dG(n−1)⌉.

The reformulation yields a degree heterogeneity measure between values of 0 for extreme cases like the null or complete graph, and 1, the maximum value. The advantage of the new measure is the normalization with respect to feasible bounds, given density and network size.

The second large subgroup base their definition of heterogeneity on the deviation of the node degrees from the mean degree in a network as described in the next paragraph. Those measures only concern degree heterogeneity.

##### 4.1.1.2. Statistic of interest: mean degree

Another concept is to measure the deviation from the mean degree as a representation of the degree heterogeneity in a graph. The higher the dispersion, the more heterogeneous the degrees. Snijders [16] favours this approach, because it considers not only the differences in relation to one most central node, but differences in all degree pairs. In particular, in real-world networks there can be not only more than one peripheral node, but also more than one central node, not to mention the nodes between the central and peripheral node(s). Therefore, Snijders [16] suggests the commonly used variance of degrees to measure degree heterogeneity or dispersion, in particular along with the graph density as an equivalent to mean and variance as descriptive statistics for numerical data. The measure is:


(4.8)HS(G):=n−1∑i=1n(ki−k¯)2,(4.9)=n−1∑i=1nki2−k¯2.

Here, the scaling constant is simply $c=n^{-1}$, the square function g and the mean function h. Like the previous approaches, Snijders [16] provides normalized versions with respect to null models and maximal possible values. The heterogeneity measure normalized with respect to the maximum value is defined as:


(4.10) $$H_{{\text{S}}^{\prime }}(G)=\sqrt{\frac{H_{\text{S}}(G)}{\max_{G^{\prime }:|V|=n,d_{G^{\prime }}=d_{G}}H_{\text{S}}(G)}}\in [0,1].$$

The minimum value of this measure is clearly zero in case of a regular graph. Snijders [16] shows that the other extreme is reached in case of graphs that consist of a group of central nodes, a group of peripheral nodes and one in between node, where [16] derive the sizes of each group so that the maximum variance is reached.

The most important advantage is that it captures if more than one node is central, unlike the Freeman [20] approach where only the most central node is considered. Furthermore, the usage of the mean as the statistic of interest has the advantage that the measure accounts for the existence of more than one high degree node, contrary to the case where only the most central node, respectively the node with the highest degree, is used as the reference point. The most important disadvantage is that, as all degree heterogeneity measures, it can fail to capture structural variations in a network owing to the focus on degree variability.

In general, similar versions of equation (4.8) are possible that capture the dispersion of the degrees [16]. For all on ${\mathbb{N}}_{\geq 0}$ monotonically non-decreasing convex functions ϕ(x) [16] suggest as general measures:


(4.11) $$\begin{array}{lcr} & H_{\text{S}}(G)=n^{-1}\sum_{i}\phi (k_{i})-\phi (\bar{k}). & \end{array}$$

The last measure of this group, also a degree heterogeneity measure, was developed by [6] and captures the extent to which individual node degrees deviate from the mean degree. It quantifies heterogeneity by summing the absolute differences between each node degree and the average degree of the graph:


(4.12) $$H_{\text{N}}(G):=\sum_{i=1}^{n}|k_{i}-\bar{k}|.$$

Therefore, the scaling parameter is c≡1, the function g is the absolute value function and h is the mean function. They mainly focus in their work on finding and exploring the tightness of upper and lower bounds for different classes of graphs. In particular, they consider star and complete bipartite graphs.

### 4.1.2. Adjacent pairwise dispersion aggregation

The next class measures graph heterogeneity by aggregating pairwise degree differences of connected nodes:


(4.13) $$c\sum_{\{i,j\}\in E}g(f(k_{i})-f(k_{j})),$$

where c again is a scaling factor and g a convex function with minimum obtained in zero for emphasizing larger differences and down-weight smaller ones. Here, f is a function of the node degrees, which usage can additionally account for relative differences between node degrees. That means a fixed difference of amount x between two small degree nodes is accounted for differently than the same difference of x between two high degree nodes, when for example choosing $f(k)=k^{-\frac{1}{2}}.$

This group contains the widely known Albertson graph heterogeneity measure [30]. The idea of [30] is that local heterogeneities (called ‘edge imbalance’) can be aggregated to create an overall heterogeneity measure, the ‘graph irregularity’, for the whole graph. They sum the local heterogeneity, measured as absolute differences in degrees between a pair of nodes, to an overall measure:


(4.14) $$H_{\text{A}}(G):=\sum_{\{i,j\}\in E}|k_{i}-k_{j}|,$$

where the scaling factor is constant 1, the convex function g equals the absolute value function and the node degree function f is the identity function.

The smallest value of 0 is reached if all nodes have the same degree, which is the case for a regular graph. Estrada [18] says that the measure reaches its highest value for a certain type of complex graph. This graph is made up of three parts: first, a closely connected group of points, second, a group of points where none are connected, and third, some connections between a point in the first group and a point in the second group [31].

Jacob *et al*. [10] point out that the aggregation of all differences does not account for differences in topologies in networks. In particular, the maximum value of the measure is not always reached for star graphs as desired by some researchers [13,18].

Another possible disadvantage of the measure is that it is only meaningfully applicable to connected graphs and it does not fulfil the definition of an irregularity measure [9]. In case of an unconnected graph with regular components the measure assigns the value zero, independent of the actual variety or uniformity of the degree value in each component. Boaventura-Netto [9] tackles the problem and derives an extended measure of the Albertson measure, that includes an additional correction parameter ξ. The proposed measure enables the assignment of a non-zero value to graphs that consist of regular, disconnected subgraphs.

The second graph heterogeneity measure in the class of adjacent pairwise dispersion aggregation approaches was developed by [18] and captures, like the Albertson measure, local discrepancies in degrees. They conclude that in heterogeneous networks nodes with high degrees must exist, which further must be connected to low degree nodes, including therefore an topological aspect in their approach. They construct a local measure of irregularity on individual edges and then aggregating these measures to obtain a measure for the entire network. They define local irregularity on edges as $I_{ij}:=(f(k_{i})-f(k_{j}){)}^{2}$ with $f(k_{i})=k_{i}^{-1/2}=\frac{1}{\sqrt{k_{i}}}$ and build their measure through aggregation of the local, relative irregularities on edges:


(4.15)HE(G):=∑{i,j}∈EIij,(4.16)=∑{i,j}∈E(ki−1/2−kj−1/2)2,(4.17)=n−21R−1/2.

Here, the scaling factor again equals 1, the convex function corresponds to the square function and the node degree function f is the inverse of the square root. Although the authors point out that any function can be chosen for f, they opt for the inverse of the square root as it allows them to express the measure using the quadratic form of the network’s Laplacian matrix and further to the Randić Index ${\;}^{1}R_{-1/2}$ [18].

The authors consider graphs that have a Poissonian degree distribution as almost regular, since the degrees are close to the average degree (in regular graphs all degrees correspond to the average degree) [18]. Contrary to this, they consider graphs with scale-free degree distribution that show a high probability for degrees that are very different (higher) than the mean degree as heterogeneous, because they show a higher deviation of the degrees from the mean degree. Based on these assumption the extremal cases for the measure are regular graphs for the minimum and star-like graphs for the maximum value. The normalized version with respect to the bounds for star-like and regular, connected graphs of their measure is:


(4.18)HE`(G)=n−21R−1/2n−2n−1,(4.19)=∑i,j∈E(ki−1/2−kj−1/2)2n−2n−1,

which leads to a graph heterogeneity measure with values between 0 (homogeneous) and 1 (heterogeneous).

One advantage is that this measure is independent of the degree distribution and a comparison of graph heterogeneity values between networks with different degree distributions is possible. Moreover, the usage of the Randić Index enables the spectral representation and projection that reflects the different heterogeneity values in networks following the ER and Barabási–Albert (BA) model.

### 4.1.3. Pairwise dispersion aggregation

The next class measures degree heterogeneity by aggregating pairwise degree differences of all nodes:


(4.20) $$\begin{array}{lcr} & c\sum_{i<j\in V}g(f(k_{i})-f(k_{j})). & \end{array}$$

Again, c is a scaling factor and g a convex function with minimum obtained in zero for emphasizing larger differences and down-weight smaller ones. f is a function of the node degrees, which usage can additionally account for relative differences between node degrees. That means a fixed difference of amount x between two small degree nodes is accounted for differently than the same difference of x between two high degree nodes, when for example choosing $f(k)=k^{-\frac{1}{2}}.$

The main difference between this measure and adjacent pairwise dispersion aggregation measures in §4.1.2 is that the latter considers degree differences only between connected nodes, capturing local topological irregularities and neighbourhood structure. By contrast, the global pairwise measure considers all node pairs, reflecting the overall degree distribution without local connectivity information. Thus, the adjacent pairwise version is a graph heterogeneity measure, able to capture clustering and local patterns, while the global pairwise version is a degree heterogeneity measure focused on degree variability across the entire network.

For example, in a uniformly connected network where all nodes share similar connectivity, both the degree differences between neighbouring nodes and those computed across all node pairs remain small. By contrast, in a network with an uneven topology, with dense clusters and sparsely connected regions, the global measure of degree differences over all nodes is high because it reflects the wide range of connectivity across the entire graph. However, within the dense clusters, neighbouring nodes tend to all have high degrees, so the local degree differences remain small within those regions.

Thus I define the adapted Albertson and Estrada measure (equations (4.14)—(4.15)) accordingly:


(4.21) $$H_{{\text{A}}_{\text{p}}}(G):=\sum_{i<j\in V}|k_{i}-k_{j}|$$

and


(4.22) $$\begin{array}{lcr} & H_{{\text{E}}_{\text{p}}}(G)=\sum_{i<j\in V}(k_{i}^{-1/2}-k_{j}^{-1/2}{)}^{2}. & \end{array}$$

### 4.2. Expected difference

The approaches in this class transfer the idea of the Gini coefficient from economics to measure heterogeneity in networks. Researchers adopt the approach to network degrees and define heterogeneity as the inequality of degrees. Gini developed several versions of the coefficient to measure income inequality [32]. Most commonly known is the version visualizable by the Lorenz curve, which displays on the *x*-axis the cumulative proportion of people from lowest to highest income and on the *y*-axis the cumulative proportion of income earned [15]. In case of absolute equality the curve corresponds to the bisectrix of the first quadrant. The higher the inequality, the more it falls below the bisectrix. The Gini coefficient is defined as the ratio between the area between the bisectrix and the Lorenz curve, divided by the complete area between the bisectrix and the *x*-axis [15]. This ratio is a value between 0, the case of absolute equality (homogeneity), and 1, the other extreme of absolute inequality (heterogeneity).

The value can be calculated as one half of the relative mean difference, which is one half of the expected value of the absolute difference of two discrete, identically and independently distributed random variables with discrete uniform probability distribution P(k), scaled by the inverse of the mean.

There are two subclasses: adjacent expected difference (AED; §4.2.1) and pairwise expected difference (PED; §4.2.2). In AED, the expected value of absolute degree differences is computed only for neighbouring nodes, while in PED, it is calculated for all possible node pairs. Both approaches capture the variability of network structure similarly to the adapted Albertson (cf. equation (4.21) ) and adapted Estrada measures (cf. equation (4.22)). AED aggregates local degree differences among adjacent nodes, capturing consistent connectivity patterns within clusters (graph heterogeneity), whereas PED aggregates differences across all node pairs, reflecting overall network variability (degree heterogeneity).

#### 4.2.1. Adjacent expected difference

In this class, assumptions are as follows: first, the degree of a node is determined by the number of incident edges; and second, the degrees of two adjacent nodes are interdependent. Therefore, the degrees are conditional on the edges. Consequently, the distribution is a conditional distribution based on the network’s structure and edge dependencies. This distribution is estimated from the network data and typically does not result in a uniform distribution.

The general formula for the graph heterogeneity measure in this class is:


(4.23) $$\begin{array}{lcr} & H_{\text{AED}}(G)=c\sum_{\{i,j\}\in E}|k_{i}-l_{j}|P(k_{i},l_{j}|\{i,j\}\in E), & \end{array}$$

with the conditional distribution $P(k_{i},l_{j}|\{i,j\}\in E)$ being the probability that a randomly chosen existing edge connects nodes with degree $k_{i}$ and $l_{j}$, conditioned on the edge existing.

#### 4.2.2. Pairwise expected difference

Measures in this class assume that the degrees of nodes are independent of the edges. They calculate the expected absolute difference in degrees between any pairs of nodes by using the product of the degree probabilities, without considering any edges. This means that the probability distribution is assumed to be marginal, meaning it considers the degree distribution across all nodes, instead of being dependent on specific edges as the AED approach in §4.2.1.

The general formula for the degree heterogeneity measures in this class is the expected absolute difference in degrees between two randomly chosen nodes i and j:


(4.24) $$\begin{array}{lcr} & H_{\text{PED}}=c\sum_{i,j\in V}|k_{i}-l_{j}|P(k_{i})P(l_{j}), & \end{array}$$

where the degrees are realizations of random variables K, L∼P that are assumed to be independently and identically distributed with expected value $\bar{k}$. The sum simply is the expected value of the absolute difference: E(|K−L|), also named as mean absolute difference. In case of $c^{-1}=\bar{k}$ it is called relative expected value, or relative mean absolute difference.

The first of the two degree heterogeneity measures in this class is developed by [4] who comments that connecting the definition of heterogeneity only to ER and PL networks, like BA networks, is not sufficient. They develop a measure that is independent of the underlying theoretical distribution of a network. They choose to measure the inequality of degrees in a network by adapting the idea of the Lorenz curve and Gini coefficient, because it enables the independence from the degree distribution and the comparability of heterogeneity between networks with different theoretical distributions.

The measure $H_{\text{HW}}$ by [4] is defined as $\frac{1}{2}$ of the relative mean absolute difference of the degrees with discrete uniform distribution $P(k)=\frac{1}{n}$:


(4.25) $$H_{\text{HW}}(G):=\frac{1}{2}\frac{1}{\bar{k}}\sum_{i,j\in V}|k_{i}-k_{j}|\frac{1}{n}\frac{1}{n}.$$

This measure represents the mean inequality of connections within a network. The value ranges from 0 to 1 and the larger the value, the more heterogeneous the degrees. The authors show that 0.5 is an upper bound for exponential networks and that the value is between 0.5 and 1 for networks whose degree distribution follow an infinite PL if and only if the degree exponent is between 2 and 2.5. Their findings demonstrate that for every network characterized by a degree distribution adhering to a PL with a degree exponent exceeding 2.5, there exists an alternative network with an exponential degree distribution that shares the same $H_{\text{HW}}$ value.

Consequently linkage between the network model (ER and BA) and the heterogeneity measure is ambiguous. Even so, the measure is applicable for networks independently of their degree distribution and additionally does not require similar network statistics, like other measures when comparing different networks (for example equation (4.8) requires the same mean degree).

The second degree heterogeneity measure in this class was developed by [17]. They view homo- and heterogeneity as the range between low and high degree nodes. They are coming from the area of network controllability and hypothesize as well as test, if high degree nodes (so called hubs) have an important role for the stability of and diffusion on networks, and therefore controlling those hubs is an important part of controlling the network. Their measure is defined as the relative mean absolute difference with discrete probability distribution $P(k)=\frac{n_{k}}{n}$:


(4.26) $$H_{\text{L}}(G):=\frac{1}{\bar{k}}\sum_{i,j\in V}|k_{i}-k_{j}|P(k_{i})P(k_{j}).$$

$H_{\text{L}}$ equals zero in networks where all nodes have the same degree, e.g. random regular digraph (in- and out-degrees are fixed to $\bar{k}/2$, nodes connected randomly). The value increases in order from random regular networks, over networks following the ER model, to PL networks with decreasing degree exponent. One disadvantage is mentioned by [26], who claims that the measure fails to connect large Cayley graphs with high degrees to heterogeneity.

### 4.3. Special heterogeneity measures

One of the first graph heterogeneity measures was introduced by [13] in their work on spectra of finite graphs. They define heterogeneity as the deviation from regularity and call it defect. This measure qualifies as a graph heterogeneity measure rather than merely a degree heterogeneity measure because it uses spectral properties (the largest eigenvalue) that capture topological information about the network structure and relationships between connected nodes, not just the degree distribution. They derive bounds for the mean degree and largest eigenvalue of a finite, connected network $k_{\text{min}}\leq \bar{k}\leq {ℵ}_{1}\leq k_{\text{max}}$ and find that a network is regular if and only if the mean degree equals the largest eigenvalue. That leads to the measure:


(4.27) $$\begin{array}{lcr} & H_{\text{CS}}(G)={ℵ}_{1}-\bar{k}, & \end{array}$$

which obtains a value of zero for regular graphs and is maximum value for star graphs (up to five nodes [13]) and several other families of graphs [33].

Another measure that does not fit the classification of above, but is worth including is developed by [10]. They define degree heterogeneity as the diversity of node degrees and not their dispersion, and in particular they are not interested in diversity of the network structure. The more different degree values exist, the more heterogeneous the degrees. This leads to different extreme cases of the measure. The star network, typically labelled as having the most heterogeneous degree distribution, is classified as homogeneous in [10], since the diversity in degrees is low (only two different degree values). The network with the most heterogeneous degree distribution is here one of n nodes where all nodes, but one, have a different degree value. They define the degree heterogeneity measure as:


(4.28) $$\begin{array}{lcr} & H_{\text{J}}(G{)}^{2}=\frac{1}{n}\sum_{k_{\text{min}}}^{k_{\text{max}}}(1-P(k){)}^{2},P(k)\neq 0. & \end{array}$$

In case of completely homogeneous degrees, the measure has a value of zero and in case of complete heterogeneity, that is every possible degree exists once, the value is $1-\frac{3}{n}+\frac{n+2}{n^{3}}$, which can be approximated by $\sqrt{1-\frac{3}{n}}$ for large networks. With these bounds they define the normalized measure with respect to complete heterogeneity in a network with the same number of nodes as:


(4.29) $$\begin{array}{lcr} & H_{{\text{J}}^{\prime }}(G)=\frac{H_{\text{J}}(G)}{\sqrt{1-\frac{3}{n}}}\in [0,1]. & \end{array}$$

The biggest advantage is that only knowledge about the degree distribution of the network is necessary for this measure. Furthermore, the measure can be calculated for any type of degree distribution or network structure and in case of the normalized measure a comparison of the heterogeneity of even differently sized large networks is possible. A disadvantage of the measures that only consider the degree sequence is that it is insensitive to changes in topologies, under the fixed degree sequence. Therefore a specific value of heterogeneity can belong to very different types of graphs (which all have the same degree sequence) and the measure does not provide a unique result. The main disadvantage of the measure is that there is a tendency to homogeneity for larger networks [10]. The reason for this might not necessarily be the size of the networks, but its density. Additionally their results of the measure behaviour for fixed/varying density and network size suggests that the density is an important factor [10].

## 5. Evaluation against properties

This section evaluates how heterogeneity measure classes fulfil the properties established in §3. I present the methodology in §5.1, results in §5.2, and discussion and implications in §5.3. The analysis covers general properties, local topology features, global structural characteristics and responses to network manipulations, guiding researchers in selecting appropriate heterogeneity measures for specific analytical needs.

### 5.1. Methodology

This section presents a multi-faceted evaluation framework that examines heterogeneity measure classes across different properties and network contexts. The nature of each evaluation component naturally necessitates specific methodological approaches, ranging from theoretical analysis for property fulfilment to simulation for graph model comparison to mathematical proofs for regular graphs. These different approaches, driven by analytical requirements rather than methodological preferences, collectively provide a comprehensive assessment of how the heterogeneity measure classes perform across theoretical and practical dimensions.

#### 5.1.1. General and local property evaluation

This section describes the approach used to assess whether each heterogeneity measure class fulfils the desired general (e.g. ability to quantify heterogeneity, uniqueness) and local topology properties (e.g. inclusion of relational structure or degree excess). To determine whether a class fulfils a given requirement, I reviewed its mathematical formulation and properties as described in §4. For example, for local topology properties, I examined whether the general measure explicitly incorporates relational information about nodes, such as degree relations or degree excess.

The following section outlines the methodology for assessing heterogeneity measures across standard network topologies using simulated random graphs.

#### 5.1.2. Graph model analysis framework

This section outlines the methodology for evaluating heterogeneity measures across standard network topologies using simulated networks. I generated ER networks, where node pairs are connected with uniform probability p, resulting in a binomial degree distribution, and PL networks, which have degree distributions $P(k)∼k^{-\gamma }$, with γ as the degree exponent.

Following the simulation set-up in [17], I created networks with 1 00 000 nodes and mean degrees (λ) ranging from 2 to 50. For ER networks, the connection probability was set as $p=\frac{\lambda }{n-1}$. For PL networks, I examined degree exponents γ of 4, 3, 2.5 and 2.2, ensuring the number of edges matched the corresponding ER networks to maintain comparable mean degrees.

All simulations were performed using the sample_gnp, and sample_fitness_pl functions from the igraph [34, 35] package in R. Initial tests indicated that the heterogeneity measures showed low variability across 100 simulation runs. Therefore, I used 100 runs for each parameter setting to make sure the estimates were stable and reliable. A summary of the simulation set-up, including parameter ranges and total number of simulated networks, is provided in table 2.

**Table 2.** Summary of simulated networks.

parameter	ER networks	PL networks	details
**number of nodes (n**)	100 000	100 000	fixed for all networks
**mean degree (λ**)	2 to 50	2 to 50	same mean degree for ER and PL
**degree exponent (γ**)	—	4, 3, 2.5, 2.2	PL only
**number of simulation runs**	100	100	per parameter setting
**total networks simulated**	2500	10 000	total: 12 250

In following section, I next describe the approach for analysing how heterogeneity measures behave on regular graphs, which serve as a theoretical baseline for minimal heterogeneity.

#### 5.1.3. Regular graph evaluation

This section details the analytical approach for determining whether each heterogeneity measure assigns zero heterogeneity to regular graphs. Several researchers require that heterogeneity measures attain their extrema for specific graph types [10,18,26]. A regular network, defined by all nodes sharing the same degree, should therefore exhibit minimal heterogeneity (measure = 0) because of its uniformity. I examine whether measures belonging to each class consistently classify regular graphs as homogeneous.

I analytically examined whether the generalized formulas of the heterogeneity measures yield a value of zero for regular graphs. By substituting the properties of regular graphs into these formulas, I derived theoretical results and provided formal proofs to verify the conditions under which each measure class classifies regular graphs as completely homogeneous.

The subsequent subsection details the methodology for comparing measure responses to structurally extreme cases: star and completely diverse networks.

#### 5.1.4. Diverse-star topology comparison

The following describes the method for comparing heterogeneity measure responses to structurally extreme cases: star graphs (one central hub, all other nodes peripheral) and completely diverse graphs (nearly every node has a unique degree). These cases represent fundamentally different connectivity patterns and test measure sensitivity to structural extremes. Proofs for measure maximization are not provided.

Star networks are sparse and highly centralized, while diverse graphs have a broad degree distribution. For example, the degree sequence (1, 1, 1, 3) in a star graph reflects a single hub connected to three peripheral nodes, while (1, 2, 2, 3) in a diverse graph shows a more balanced distribution. Measures emphasizing extremes may rate star graphs as more heterogeneous, while those considering overall distribution may favour diverse networks.

Direct comparison is challenging: star graphs are inherently sparse, while diverse graphs quickly become dense as size increases, making it difficult to match edge densities and control for density effects. Identifying truly diverse networks, where each node has a unique degree, is also combinatorially difficult for larger sizes. Therefore, I restrict the analysis to the smallest non-trivial star and diverse networks with increasing order of 4 to 7 (see appendix D for network statistics and degree sequences) to gain insight into the behaviour of the measures relative to network type and network size.

Limitations include the small sample size and varying edge densities (0.29−0.67), which may confound results. Because edge density can affect degree variability and topology, differences in heterogeneity values may reflect either structural differences or density effects. Additionally, normalization of heterogeneity values across the sample does not guarantee that observed maxima represent theoretical maxima.

This analysis serves as an initial exploration; future work should examine larger and intermediate topologies and consider normalization for edge density.

Next, I outline the methodology for testing how the measures respond to network manipulation.

#### 5.1.5. Network manipulation testing

Finally, I present the methodology for testing how heterogeneity measures respond to network manipulations, including edge rewiring, uniform degree increase and network replication, corresponding to the three principles by [7] presented in §3.4. For the three dispersion aggregation classes, I derived sufficient mathematical conditions for compliance through formal analysis of their formulations, examining how components such as the convex function g, reference statistic h(K), and aggregation method influence behaviour. For other classes, general conditions could not be derived owing to mathematical complexity; instead, I constructed and analysed specific counter-examples to demonstrate non-compliance.

This combined analytical and example-based approach highlights both the general theoretical properties and the practical behaviours of heterogeneity measures in response to structural changes. The results reveal key differences in how these measures respond to network modifications, as discussed below.

With these methodologies established, I now present the results of evaluating the heterogeneity measures across the outlined network properties and structures.

### 5.2. Results

#### 5.2.1. General and local property fulfilment

My evaluation reveals that all heterogeneity measure classes successfully quantify heterogeneity according to their respective definitions and produce interpretable, unique values. These measures can be applied to various network types and sizes with one exception: only measure $H_{\text{CS}}$ proposed by [13] specifically requires networks to be connected.

My analysis shows that most heterogeneity measures require more than just the degree distribution as input. Only the measure $H_{J}$ can operate with the degree distribution alone as its primary input. All other measures, including those in the adjacent dispersion aggregation and AED classes, as well as the $H_{\text{CS}}$ measure, require additional network information beyond the degree distribution.

Regarding local topology, I find systematic differences between measure classes: first, all dispersion aggregation classes, except the global dispersion aggregation class, incorporate information about local topology by considering the relational structure of the network. Second, the global dispersion aggregation class uniquely accounts for degree excess, distinguishing it from other measure classes. Third, adjacent pairwise dispersion aggregation, pairwise dispersion aggregation and both expected difference subclasses (adjacent and pairwise) incorporate weighted or relative degree relationships in their calculations.

All measure classes successfully fulfil almost all general properties required of heterogeneity measures and only show differences regarding the degree distribution as input and all local topology properties.

Having presented the baseline properties, I next continue with the performance results across standard network models.

#### 5.2.2. Graph model heterogeneity patterns

**Figure 1.**
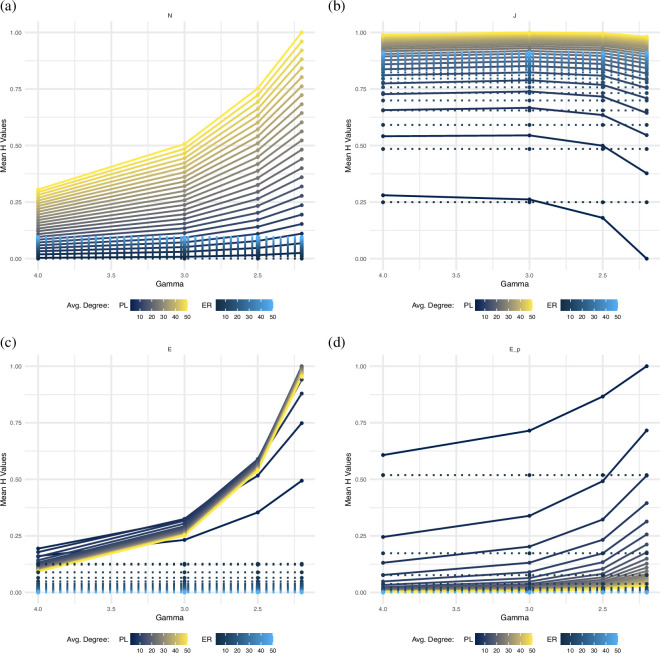
Panels show normalized heterogeneity values for simulated ER and PL networks, illustrating how heterogeneity changes with decreasing degree exponent (γ) and varying mean degrees (from 2 to 50). In general, most measures assign higher heterogeneity values to PL networks than ER networks, with differences becoming more pronounced with increasing network density. Moreover, among PL networks, most measures assign higher heterogeneity values as the degree exponent decreases. (a) This plot exemplifies the typical behavior of dispersion aggregation and expected difference measures. Measures consistently show higher heterogeneity values for PL networks than for ER networks across all *γ* values. (b) The degree heterogeneity measure *H*_J_ exhibits inconsistent behavior. Differentiation between ER and PL networks is unreliable, with reversals in heterogeneity ranking at lower *γ* values and poor distinction for networks with low mean degrees. (c) The measure assigns higher heterogeneity values to PL networks as the degree exponent decreases. For mean degrees above 20 the heterogeneity values tend to stabilize. (d) The measure _$H_{{\text{E}}_{\text{p}}}$_ mirrors the pattern in figure 1c with increasing heterogeneity for lower *γ*. For networks with mean degrees higher than 20, the values are uniformly low, lessening the differentiation between ER and PL networks.

Figure 1 presents the results of four selected^1^ heterogeneity measures ($H_{\text{N}}$, $H_{\text{J}}$, $H_{\text{E}}$ and $H_{{\text{E}}_{\text{p}}}$) across simulated ER and PL networks. Each plot shows normalized heterogeneity values scaled to the unit interval based on minimum and maximum values across all networks for each measure. These four measures represent the variety of behaviours exhibited across all heterogeneity measures: $H_{\text{N}}$ (figure 1a) represents the typical behaviour of most dispersion aggregation and expected difference measures; $H_{\text{J}}$ (figure 1b) exhibits inconsistent behaviour; and $H_{\text{E}}$ and $H_{{\text{E}}_{\text{p}}}$ (figure 1c,d) show different behaviour in differentiating network topologies at higher mean degrees.

Most heterogeneity measures consistently identify PL networks as more heterogeneous than ER networks across all parameter settings. This distinction is clearly visible in figure 1a, where the most yellow line (PL networks) consistently ranks above the most blue line (ER networks). This reflects the fundamental difference between ER networks’ uniform connectivity and PL networks’ hub-based structure.

For degree exponents of 3 and 4, several measures ($H_{\text{F}}$, $H_{\text{S}}$, $H_{\text{A}}$, $H_{\text{E}}$, $H_{{\text{E}}_{\text{p}}}$, $H_{\text{AED}}$ and $H_{\text{CS}}$) still identify PL networks as more heterogeneous, but assign similar low values to both. It is important to note that almost half of the measures, including $H_{\text{N}}$, $H_{\text{S}}$, $H_{\text{A}}$, $H_{{\text{A}}_{\text{p}}}$ and $H_{\text{PED}}$ do not effectively differentiate between PL and ER models when the networks are sparse (i.e. at low mean degree).

Analysing the effects of network parameters on heterogeneity values demonstrates distinct patterns for both degree exponent and mean degree variations.

Degree exponent effects: for almost all measures, heterogeneity values increase as the degree exponent (γ) decreases, even in sparse networks. In figure 1a,c,d, networks with γ=2.2 consistently show the highest heterogeneity values, while those with γ=4 show the lowest. This pattern reflects how smaller exponents create more extreme hub-and-spoke structures with greater connectivity differences.

Mean degree effects: the distinction between ER and PL networks becomes more pronounced with increasing mean degrees for most heterogeneity measures. However, this is not universal. The Estrada measure (figure 1c) shows values that stabilize for mean degrees 20 and higher, while the adapted Estrada measure (figure 1d) assigns similar low values to all networks with mean degree larger than 20−30 (see also figure 5 in appendix A).

While most measures exhibit consistent patterns, the degree heterogeneity measure $H_{\text{J}}$ demonstrates behaviour that deviates significantly from the general trends.

$H_{\text{J}}$ fails to consistently differentiate between ER and PL networks, even reversing its assignments for values of γ at 2.5 and lower (see figure 1b). $H_{\text{J}}$ also demonstrates an unexpected ranking pattern among PL networks. For mean degree 20, $H_{\text{J}}$ ranks heterogeneity as γ=3>γ=2.5>γ=4>γ=2.2, contradicting the pattern seen in other measures where lower exponents correspond to higher heterogeneity. Additionally, $H_{\text{J}}$ shows limited ability to distinguish networks with small mean degrees, further differentiating it from other heterogeneity measures.

#### 5.2.3. Regular graph zero-value conditions

Regular graphs, characterized by uniform node degrees, should yield a heterogeneity value of zero (indicating perfect uniformity). My analysis confirms that all examined measures assign zero to regular graphs, though through different mathematical mechanisms specific to each measure class. Table 6 in appendix B summarizes these conditions.

In the *global dispersion aggregation* class (cf. equation (4.1)), regular graphs are classified as completely homogeneous when the difference between the degree and the statistic (r−h(K)) equals one of the roots of the convex function g. The formal proposition and its proof, which derive these conditions, are provided in appendix B.

For both the *adjacent pairwise and pairwise dispersion aggregation* classes (generalized by equations (4.13)–(4.20)), the heterogeneity measures are minimized for regular graphs when the scaling factor is positive. This occurs, because the function g attains its minimum of value zero at zero, by definition.

Similar reasoning applies to the *expected difference* class (see equations (4.23)—(4.24)), where regular graphs are considered as completely homogeneous if the scaling factor is positive.

Finally, the *special measures* $H_{\text{CS}}$ and $H_{\text{J}}$ are defined such that they evaluate to zero when applied to regular graphs. For all finite, connected graphs the inequality $\bar{k}\leq {ℵ}_{1}$ holds true and in any r-regular graph, both the dominant eigenvalue ${ℵ}_{1}$ and mean degree $\bar{k}$ are equal r (Satz 2 in [13]). This equality ensures that $H_{\text{CS}}$ (cf. equation (4.27)) is zero; therefore, the measure is minimized for r-regular graphs.

For an r-regular graph G the degree distribution is defined by P(k)=1 for k=r and zero otherwise. This leads directly to a heterogeneity value of zero for the non-negative heterogeneity measure $H_{\text{J}}$ of [10], shown in equation (4.28).

These findings demonstrate that all heterogeneity measure classes (with only modest conditions) uniformly classify regular graphs as homogeneous with value zero. Every specific measure examined in this work assigns regular graphs the minimum heterogeneity value of zero, though through different mathematical mechanisms.

#### 5.2.4. Diverse-star topology differentiation

**Figure 2.**
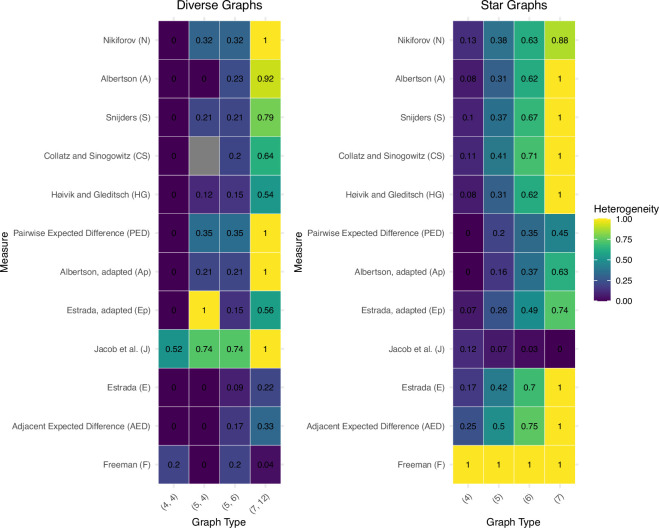
Heat maps of normalized heterogeneity values (0−1) comparing diverse graphs (left, with nearly unique node degrees) and star graphs (right, with one high-degree central node connected to all other nodes). Each row represents one of twelve heterogeneity measures, while columns represent specific graph configurations labelled by node count and edges (e.g. (7, 12): diverse graph with seven nodes, 12 edges; (7): star graph with seven nodes). Key findings include: (i) most measures assign higher values to star graphs, reflecting sensitivity to centralized structures; (ii) $H_{\text{F}}$ consistently assigns maximum values (1.0) to all star graphs; (iii) $H_{\text{J}}$ uniquely assigns maximum values to diverse graphs, particularly graph (7,12); and (iv) pairwise measures ($H_{\text{PED}}$, $H_{{\text{A}}_{\text{p}}}$, $H_{{\text{E}}_{\text{p}}}$) show inconsistent differentiation patterns, sometimes assigning maximum values to diverse graphs rather than stars. Most measures demonstrate increasing heterogeneity values with network size for star graphs, while diverse graphs produce more variable assessments across measures. These contrasting behaviours demonstrate how different mathematical conceptualizations of heterogeneity can lead to fundamentally different network assessments.

Figure 2 presents normalized heterogeneity values for 12 measures across two contrasting network topologies: star graphs (centralized structure with one dominant node) and diverse graphs (distributed structure with nearly unique degrees). My analysis reveals three key findings: first, most measures assign higher heterogeneity values to star graphs than diverse graphs. By contrast, assessments of diverse graphs are more variable and lack the consistent, size-dependent trends seen in star graphs. Second, measure $H_{\text{F}}$ consistently identifies star graphs as highly heterogeneous across all sizes. Third, the degree heterogeneity measure $H_{\text{J}}$ uniquely recognizes diverse graphs as more heterogeneous, contrary to other measures’ assessments.

Heterogeneity values for star graphs show clear trends as network size increases. Most measures assign values that increase with graph size, reflecting the growing structural irregularity as the central node’s dominance becomes more pronounced in larger networks.

The measures $H_{\text{J}}$, $H_{\text{E}}$, $H_{\text{AED}}$ and $H_{\text{F}}$ effectively differentiate between diverse and star graphs. Specifically, $H_{\text{F}}$ consistently assigns maximum values (1.0) to all star graphs while giving low values (≤0.2) to diverse graphs. Conversely, $H_{\text{J}}$ consistently assigns high values to diverse graphs (with a maximum of 1.0 to graph (7,12)) and progressively lower values to star graphs. Both $H_{\text{E}}$ and $H_{\text{AED}}$ show clear, increasing trends with network size for star graphs, reflecting their sensitivity to increasingly centralized structures.

All pairwise measures ($H_{\text{PED}}$, $H_{{\text{A}}_{\text{p}}}$, $H_{{\text{E}}_{\text{p}}}$) show increasing trends for star graphs but exhibit high variability for diverse graphs without consistent patterns. Notably, $H_{\text{PED}}$ and $H_{{\text{A}}_{\text{p}}}$ both assign their maximum value (1.0) to the diverse graph (7,12), rather than to any star graph. $H_{{\text{E}}_{\text{p}}}$ uniquely assigns its maximum value (1.0) to the diverse graph (5,4), showing distinct behaviour from the other measures.

The remaining measures ($H_{\text{N}}$, $H_{\text{A}}$, $H_{\text{S}}$, $H_{\text{CS}}$, $H_{\text{HG}}$) generally assign higher values to star graphs than diverse graphs and provide good discrimination between graph types. Notably, the diverse graph (7,12) receives relatively high values from these measures, comparable with those assigned to larger star graphs.

The heat maps in figure 2 highlight significant variation in how effectively different measures distinguish between these contrasting topologies. While most measures assign higher values to star graphs, suggesting greater sensitivity to centralized structures, $H_{\text{J}}$ provides an opposite assessment, assigning higher values to diverse graphs. This contrasting behaviour reflects fundamental differences in how heterogeneity is mathematically conceptualized across measures, with some focusing on centralization structures and others on degree diversity.

#### 5.2.5. Network manipulation responses

**Figure 3.**
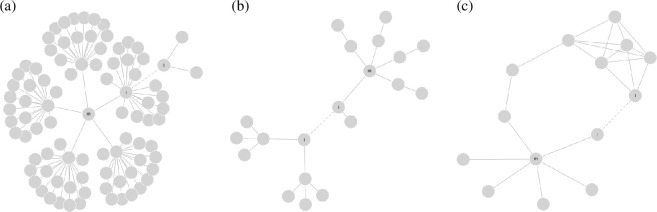
Networks illustrating counter-examples where heterogeneity measures increase after rewiring, violating the transfer principle (see table 4 for corresponding numerical values). Dashed lines indicate the new connections. (a) Counter-example for the graph heterogeneity measures *H*_A_, *H*_E_, *H*_EP_ and *H*_AED_. (b) Counter-example for the graph heterogeneity measure *H*_PED_ and the degree heterogeneity measures *H*_J_. (c) Counter-example for the graph heterogeneity measures *H*_CS_.

**Figure 5.**
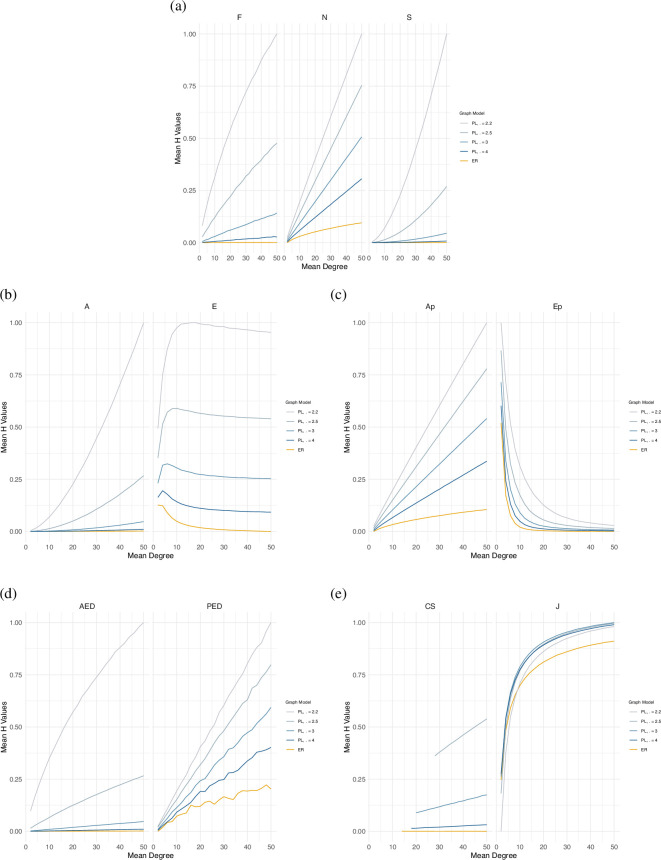
Mean heterogeneity values for different measures against mean degree ($\bar{k}=2,\ldots ,50$). The measures are calculated for simulated random networks whose node degrees follow the ER graph model and scale-free networks with decreasing degree exponent γ∈{4,3,2.5,2.2} (scale-free (PL) graph model). (a−e) show the results for measures belonging to the five different classes, scaled to the unit interval. The key findings are firstly, all measures, but the Jacob measure assigns increasing heterogeneity values beginning with the lowest for ER models and then for PL models with decreasing degree over all mean degrees. Secondly, the distinction between ER and PL models is less clear for sparse graphs, and mostly becoming more obvious for denser networks. Note, that the results for the $H_{\text{CS}}$ measure could only be obtained for sufficiently dense graphs, so that they are connected. (a) Global dispersion aggregation measures; (b) adj. pairwise dispersion aggregation measures; (c) pairwise dispersion aggregation measures; (d) expected difference measures; and (e) special measures.

The evaluation of heterogeneity measures against network manipulation principles revealed that only 1 of 12 measures ($H_{\text{S}}$) satisfies all three network manipulation principles, while two ($H_{\text{AED}}$ and $H_{\text{CS}}$) comply with none. The addition principle shows highest compliance (10 out of 12 measures), with transfer and replication principles each satisfied by only 2 out of 12 measures. Most measures (8 out of 12) comply with exactly one principle, typically addition. Table 3 summarizes these findings, with non-compliance cases illustrated in figure 3 and table 4. For dispersion aggregation measures, I derived sufficient conditions for compliance, detailed in table 7 (appendix C).

**Table 3.** Compliance of heterogeneity measure classes with network principles.

measure class	measure	transfer	addition	replication
global dispersion aggregation	$H_{\text{N}}$	×	✓	×
	$H_{\text{S}}$	✓	✓	✓
	$H_{\text{F}}$	×	✓	×
	$H_{\text{HG}}$	×	✓	×
adjacent pairwise dispersion	$H_{\text{A}}$	×	✓	×
	$H_{\text{E}}$	×	✓	×
pairwise dispersion	$H_{{\text{A}}_{\text{p}}}$	✓	✓	×
	$H_{{\text{E}}_{\text{p}}}$	×	✓	×
expected difference	$H_{\text{AED}}$	×	×	×
	$H_{\text{PED}}$	×	✓	×
special measures	$H_{\text{J}}$	×	✓	✓
	$H_{\text{CS}}$	×	×	×

**Table 4.** Heterogeneity values before and after edge rewiring.

measure	before rewiring	after rewiring	figure reference
$H_{\text{A}}$	1342.00	1350.00	3a
$H_{\text{E}}$	46.30	46.60	3a
$H_{\text{AED}}$	15.40	15.50	3a
$H_{\text{N}}$	17.6	17.6	3b
$H_{{\text{E}}_{\text{p}}}$	15.40	15.70	3b
$H_{\text{J}}$	0.38	0.41	3b
$H_{\text{PED}}$	1.47	1.70	3c
$H_{\text{CS}}$	1.47	1.49	3c

*Global dispersion aggregation* measures (cf. equation (4.1)) demonstrate distinct compliance patterns. All four measures, $H_{\text{S}}$ (cf. equation (4.8)), $H_{\text{N}}$ (cf. equation (4.12)), $H_{\text{HG}}$ (cf. equation (4.2)) and $H_{\text{F}}$ (cf. equation (4.5)), comply with the addition principle. However, only $H_{\text{S}}$ additionally satisfies both the transfer and replication principles, making it uniquely compliant with all three principles.

For the transfer principle, compliance requires the network statistic to remain unchanged during rewiring ($h(K)=h(K^{\prime })$) with specific functional conditions satisfied. The addition principle compliance occurs when network statistics increase proportionally with node degrees or when g is monotone increasing and $h(K^{\prime })>h(K)$. For the replication principle, compliance occurs only when the replication process maintains the network statistic and scaling factors satisfy $x=c/c^{\prime }$.

(*Adjacent*) *pairwise dispersion* measures exhibit similar compliance patterns. Most breach the transfer principle, as shown in table 4: $H_{\text{A}}$ increases from 1342 to 1350, $H_{\text{E}}$ from 46.3 to 46.6 and $H_{{\text{E}}_{\text{p}}}$ from 15.4 to 15.7. Only the pairwise measure $H_{{\text{A}}_{\text{p}}}$ complies with the transfer principle. All measures in both classes comply with the addition principle but fail the replication principle, with adjacent measures scaling linearly and adjacent pairwise measures scaling quadratically with the replication factor.

*Expected difference* measures $H_{\text{PED}}$ (cf. equation (4.24)) and $H_{\text{AED}}$ (cf. equation (4.23)) breach the transfer principle, with $H_{\text{PED}}$ increasing from 1.47 to 1.7 (figure 3c) and $H_{\text{AED}}$ from 15.4 to 15.5 (figure 3a) after rewiring. $H_{\text{PED}}$ complies with the addition principle while $H_{\text{AED}}$ does not. Neither satisfies the replication principle.

*Special measures* $H_{\text{J}}$ (cf. equation (4.28)) and $H_{\text{CS}}$ (cf. equation (4.27)) breach the transfer principle, with $H_{\text{J}}$ increasing from 0.38 to 0.41 and $H_{\text{CS}}$ from 1.47 to 1.49. $H_{\text{J}}$ uniquely satisfies the replication principle because it depends solely on the degree distribution (unchanged during replication), while $H_{\text{CS}}$ breaches all principles.

Having established the empirical compliance patterns of heterogeneity measures across the classes, I now interpret these findings to understand their theoretical foundations and practical implications.

### 5.3. Discussion

This section presents a multifaceted evaluation of heterogeneity measures through four complementary analyses. The graph model analysis reveals how measures distinguish between uniform ER networks and heterogeneous PL networks with decreasing degree exponents. The regular graph analysis confirms the mathematical mechanisms by which measures assign zero values to regular networks. The diverse-star comparison examines measure sensitivity to contrasting network architectures that embody different organizational principles. Finally, the network manipulation analysis investigates measure compliance with principles of edge transfer, degree addition and network replication. Collectively, these evaluations demonstrate how different mathematical formulations of heterogeneity lead to distinct behaviours across network contexts, highlighting the importance of measure selection based on specific research requirements.

#### 5.3.1. Graph model heterogeneity interpretation

The simulation study reveals that most heterogeneity measures successfully distinguish between the uniform structure of ER networks and the heterogeneous architecture of PL networks, while also ranking PL networks according to their degree exponent, though with varying sensitivities to network parameters.

ER networks exhibit uniform connectivity patterns that remain independent of changes in mean degree, typically yielding lower heterogeneity values reflecting their homogeneity. By contrast, PL networks display heterogeneous connectivity. Decreasing the degree exponent in PL networks produces a heavier tail in the degree distribution, resulting in increased probability of hubs within the network. This leads to a greater contrast between a few highly connected nodes and many sparsely connected ones. Increasing the mean degree, while maintaining the number of nodes fixed, leads to an increase of degree for both hubs and less connected nodes. Although the hubs’ absolute degree increases, the relative difference between them and all other nodes remains the same, because the entire degree distribution is shifted upwards rather than being reshaped.

As expected, degree and graph heterogeneity measures generally assign higher values to PL graphs than to ER graphs, reflecting their greater structural variability. Within PL networks, decreasing exponents correspond to increasing measure values for most metrics, capturing the greater degree variability and hub formation characteristic of networks with heavier-tailed distributions.

The degree heterogeneity measures $H_{\text{N}}$ and $H_{\text{S}}$ capture the uniform variation in ER graphs and the increasing variation in degrees that comes from the heavier tail in PL networks with smaller degree exponents, by measuring the variation around the mean degree.

The degree heterogeneity measure $H_{\text{J}}$ inconsistently differentiates between ER and PL networks as the degree exponent decreases. This occurs because of competing effects: decreasing γ increases highly connected nodes (individually contributing more to heterogeneity), while simultaneously concentrating the distribution among fewer high-degree nodes (reducing overall degree diversity). Although adding new hubs increases individual contributions, $H_{\text{J}}$ merely recognizes new degrees without adequately weighting their magnitude relative to other nodes. Thus, despite higher individual contributions, the reduced diversity in degree types ultimately lowers the measure’s value.

The degree heterogeneity measure $H_{\text{F}}$ assesses differences between node degrees and the maximum degree node. As PL exponents decrease, the increasing probability of hubs leads to greater differences between maximum degree and other nodes, effectively capturing hub formation.

Measures based on degree differences ($H_{\text{A}}$, $H_{\text{AED}}$, $H_{\text{PED}}$) capture structural variations by focusing on local and global degree disparities. These measures are particularly sensitive to the growing presence of hubs in PL networks with decreasing exponents. As the exponent decreases, the disparity between highly connected hubs and low-degree nodes becomes more pronounced, resulting in increased measure values.

The degree heterogeneity measures $H_{{\text{A}}_{\text{p}}}$ and $H_{\text{PED}}$ are pairwise measures and have heterogeneity values dependent on degree differences between all nodes. In networks with many low-degree nodes, differences between these nodes contribute little to overall heterogeneity. However, comparisons between low-degree nodes and high-degree hubs produce significantly larger differences. As degree exponents decrease, hub prevalence increases, amplifying the disparity between sparsely connected nodes and highly connected hubs, leading to greater contribution from these larger differences and increasing overall heterogeneity.

The degree heterogeneity measure $H_{{\text{E}}_{\text{p}}}$ offers a unique perspective by dampening the contribution of hubs relative to low-degree nodes through a square root transformation. In PL networks with lower degree exponents where hubs become more prevalent, the absolute differences in degrees increase. However, the measure dampens these differences through its transformation, reducing the extreme contributions from high-degree hubs. As a result, $H_{{\text{E}}_{\text{p}}}$ tends to perceive the overall network structure as more homogeneous than other measures might suggest.

Across all measures, the graph heterogeneity measure $H_{\text{E}}$ demonstrates unique stability across changing mean degrees, suggesting it may be particularly suitable to reveal connectivity disparities among moderate-degree nodes that would otherwise be overshadowed by hubs. By contrast, most other measures showed sensitivity to mean degree changes, highlighting the importance of controlling for network density when comparing heterogeneity across networks.

For degree exponents of 3 and 4, several measures ($H_{\text{F}}$, $H_{\text{S}}$, $H_{\text{A}}$, $H_{\text{E}}$, $H_{{\text{E}}_{\text{p}}}$, $H_{\text{AED}}$ and $H_{\text{CS}}$) assign similar low values to both PL and ER networks while still ranking PL networks as more heterogeneous. This observation aligns with previous research suggesting that for PL networks with exponents above 2.5, there exists an ER network with equivalent heterogeneity values [4].

While most measures successfully differentiate between network structures, their sensitivity varies substantially with network parameters. The divergent behaviour of $H_{\text{J}}$ compared with other measures highlights how different conceptualizations of heterogeneity can lead to dramatically different conclusions about the same network. These findings underscore the importance of selecting heterogeneity measures based on specific research questions and theoretical frameworks.

This study demonstrates that degree and graph heterogeneity measures capture complementary aspects of network organization. Researchers should consider employing multiple measures to gain comprehensive insights into network structure, particularly when analysing complex real-world networks where heterogeneity manifests in various forms. Future work might explore how these measures perform on empirical networks with more complex structural properties beyond the idealized ER and PL models examined here.

#### 5.3.2. Regular graph zero-value mechanisms

The analysis confirms that all measures return a value of zero for regular graphs. However, this common outcome masks important distinctions in the underlying theoretical mechanisms by which different measure classes arrive at this conclusion.

Measures in the adjacent pairwise dispersion aggregation and expected difference classes (cf. equations (4.13)–(4.23)) reach zero through direct mathematical consequences of their definitions. These measures are constructed directly on differences between node degrees or transformations of these differences. Because regular graphs are defined by uniform node degrees (where all nodes have identical degrees), these differences inherently vanish, leading to a zero value by mathematical construction.

By contrast, measures in the global dispersion aggregation class (cf. equation (4.1)) employ a fundamentally different, centrality-based perspective. These measures also assign a zero value for regular graphs, but only under specific mathematical conditions. As proved in appendix C, for these measures to reach zero, the difference between the network’s node degree (r) and the key statistic h(K) must exactly match one of the roots of the convex function g. This additional requirement reflects their underlying centrality view rather than a direct focus on degree differences.

This distinction highlights that while the outcome is the same, the theoretical approaches and built-in conditions of these measures differ.

An important limitation emerges from this analysis: the current set of heterogeneity measures cannot distinguish between different types of regular graphs that might vary in other structural properties. For example, two regular graphs with identical degree distributions but different patterns of edge arrangement (evenly distributed versus clustered connections) would receive identical heterogeneity scores of zero. This limitation suggests that researchers interested in capturing more nuanced aspects of homogeneity beyond degree regularity may need to employ complementary measures that can detect differences in edge distribution patterns or other structural properties.

#### 5.3.3. Diverse-star topology sensitivity

Heterogeneity measures vary in their ability to distinguish between star-like and diverse network topologies, with only some measures ($H_{\text{E}}$, $H_{\text{AED}}$, $H_{\text{F}}$ and $H_{\text{J}}$) clearly differentiating these structures while others show limited sensitivity despite the contrasting organizational principles these topologies represent.

The expected behaviour of the heterogeneity measures can be summarized as follows: first, graph heterogeneity measures that quantify local degree irregularities are expected to recognize star graphs, which exhibit high local degree differences, as heterogeneous. Second, measures capturing deviations from a most central point in the network should assign high values to star graphs because of their extreme centralization, while diverse graphs might be assigned lower scores owing to their more distributed nature. Third, degree heterogeneity measures, aiming to capture variability in degrees, should distinguish both topologies well.

Measures such as the graph heterogeneity measures $H_{\text{E}}$ and $H_{\text{AED}}$ that incorporate local degree differences effectively recognize the structural differences between star and diverse networks. Specifically, $H_{\text{E}}$ assigns high values to star graphs because of the pronounced degree difference between the central hub and its neighbours. Similarly, $H_{\text{AED}}$ distinguishes between the two topologies well while capturing degree irregularities.

Degree heterogeneity measures $H_{\text{F}}$ and $H_{\text{HG}}$ aim to quantify deviations from a most centralized point in the network. $H_{\text{F}}$ employs normalization against the theoretical maximum possible centralization, using perfect star topology as the reference point. This normalization approach explains its distinctive binary classification behaviour observed in the heat map in figure 2, where star graphs consistently achieve maximum heterogeneity scores (1.0), while diverse graphs receive substantially lower values (≤0.2). $H_{\text{HG}}$ measures the relative deviations from the most central node without explicit normalization against a theoretical maximum, which leads to increasing values for increasing star graphs as well as a good distinction from diverse graphs.

The set of degree heterogeneity measures aim to capture variability in node degrees across the network. $H_{\text{J}}$ successfully distinguishes between diverse and star networks by recognizing the broad range of degrees in diverse graphs. $H_{\text{N}}$ and $H_{\text{S}}$ are more focused on the deviation around the mean degree, and given the small network sizes with limited variation around the mean, these measures do but not strongly differentiate between the two topologies. Measures which are based on pairwise irregularities ($H_{\text{PED}}$, $H_{{\text{A}}_{\text{p}}}$, $H_{{\text{E}}_{\text{p}}}$) exhibit more inconsistent results. One reason is that in star graphs irregularities involving the central node are few and most degree pair irregularities yield zero. For diverse graphs with nearly unique degrees, more pairs have non-zero differences. This can lead to maximum heterogeneity values in diverse graphs rather than star graphs in some cases.

In future work, it would be valuable to relax the strict definitions of the extreme cases used in the current study. Instead of focusing solely on networks that are completely star-like or entirely diverse, exploring networks that exhibit high, but not absolute, diversity could provide a more realistic image of many real-world systems. By considering such intermediate cases, one could examine how heterogeneity measures perform across a broader spectrum of network configurations. Additionally, larger networks and adjustments such as normalizing by edge density or applying theoretical bounds may also provide further insights into how these measures capture centralized versus distributed network structures.

#### 5.3.4. Compliance with network manipulation principles

This discussion examines how heterogeneity measures respond to three fundamental network principles proposed by [7]: transfer, addition and replication. By analysing mathematical properties across measure classes, I discuss why certain measures comply with these principles while others exhibit counter-intuitive behaviours.

The transfer principle states that network heterogeneity should decrease when an edge is rewired from a higher-degree node to a lower-degree node (while maintaining the condition that the target node’s degree remains higher than the originating node’s degree). While degree heterogeneity measures are directly influenced by node degrees, their response depends on how variations are quantified. Graph heterogeneity measures present more complex behaviour, particularly those based on centrality or ones that capture both degree distribution and topological structure.

The addition principle requires that heterogeneity should not increase when all node degrees are uniformly increased. For measures based on absolute differences (like variation around the mean), values should remain constant, while measures of relative differences should decrease. Degree heterogeneity measures would be expected to satisfy this principle because of their direct dependence on degree distribution scaling. Graph heterogeneity measures incorporating the networks structure are also expected to reflect changes, as increasing all degrees changes the network’s topology, but the direction of change depends on how nodes are connected.

The replication principle holds that heterogeneity values should remain unchanged when a network is replicated (whether by integer or non-integer multiples). For integer replication, the degree distribution remains unchanged, while non-integer replication requires proportional scaling of node counts. Measures focusing solely on degree distribution are expected to comply, while those incorporating structural properties may reflect the topological changes introduced by creating disconnected components.

*Global dispersion aggregation* measures quantify heterogeneity by comparing node properties to a network-wide statistic. Their response to the three principles varies based on their mathematical formulation and the network statistics they employ.

Regarding the transfer principle, these measures (cf. equation (4.1)) respond differently to edge rewiring based on their reference statistic (h(K)) and aggregation function (g). Mean-based measures exhibit contrasting behaviours: $H_{\text{N}}$ (cf. equation (4.12)) quantifies absolute deviations, detecting changes only when rewiring causes degrees to cross the mean threshold, while $H_{\text{S}}$ (cf. equation (4.8)) uses squared differences that ensure all deviations contribute positively without cancellation, fully complying with the principle. Maximum-based measures $H_{\text{HG}}$ (cf. equation (4.2)) and $H_{\text{F}}$ (cf. equation (4.5)) breach the principle when rewiring occurs among non-maximum degree nodes, as changes in nodes’ degrees simply cancel out without affecting the reference statistic. Their sensitivity depends primarily on whether the rewiring affects the maximum degree node.

When considering the addition principle, these measures satisfy its requirements because the relative differences among node degrees remain unchanged after uniform increase. This consistent behaviour contrasts with their variable responses to the transfer principle.

For the replication principle, these measures comply only if the network statistic remains unchanged (i.e. $h(K)=h(K^{\prime })$) and the relationship between scaling factor (x) and measure constants satisfies $x=c/c^{\prime }$. Otherwise, they scale with replication, like $H_{\text{HG}}$ and $H_{\text{N}}$. This scaling behaviour results from the linear addition of nodes during replication. For example, $H_{\text{F}}$ decreases when replicated because while the degree differences within each component remain unchanged, the reference value (maximum possible difference) increases substantially, reducing the normalized measure. Detailed proofs are provided in appendix C.

*Adjacent and pairwise dispersion aggregation* measures quantify heterogeneity through local and global degree differences, showing varied responses to network modifications across the three principles.

Regarding the transfer principle, these measures exhibit counter-intuitive behaviour. While one might expect them to satisfy this principle (since rewiring should reduce degree differences between connected nodes), they often breach it because of neighbourhood effects. For measures $H_{\text{A}}$ (cf. equation (4.14)) and $H_{\text{E}}$ (cf. equation (4.15)), graph heterogeneity can increase even when the rewired edge’s degree difference decreases, as shown in figure 3a. This occurs when the node losing an edge connects to high-degree neighbours while the node gaining a connection links to low-degree neighbours, amplifying overall heterogeneity.

For degree heterogeneity measures aggregating across all node pairs, compliance with the transfer principle depends critically on the choice of functions and constants. While the Albertson measure (adapted for all pairs) fulfils the principle (proof in appendix C), the adapted Estrada measure (cf. equation (4.22)) with parameter c=1, functions $g(\cdot )=\cdot^{2}$ and $f(\cdot )=\cdot^{-0.5}$ can increase after rewiring. For the network in figure 3b, this measure increases from $H_{{\text{E}}_{\text{p}}}(G)=15.4$ to $H_{{\text{E}}_{\text{p}}}(G^{\prime })=15.7$. This occurs because the inverse square root function down-weights high-degree nodes while the quadratic function amplifies larger differences, allowing small increases in lower-degree node differences to outweigh decreases from higher-degree nodes.

For the addition principle, these measures generally comply as expected, since uniformly increasing node degrees preserves pairwise differences. This holds particularly for measures using the identity function as f (proof in appendix C).

Under replication, both measure classes breach the principle by scaling with network replication. Adjacent pairwise measures scale linearly with the replication factor, as the same sum of differences is computed for each network copy and aggregated over the edge set. Pairwise measures scale quadratically, as they sum over every combination of nodes from the original network and its copies, causing the measure to grow as the square of the replication factor.

*Expected difference* measures adapt the Gini coefficient concept to quantify how much nodes differ in their degrees on average. These measures display varied behaviours across the three network principles.

Regarding the transfer principle, measures $H_{\text{AED}}$ and $H_{\text{PED}}$ (cf. equations (4.23)–(4.24)) behave differently depending on degree distribution assumptions. Under uniform distribution, they reduce to scaled versions of either the Albertson measure or its adaptation. While the adapted version complies with the principle (proof in appendix C), the original measure breaches it (see figure 3a). Under non-uniform distributions, both measures can breach the principle as rewiring affects not only absolute differences but also joint probability distributions. This complexity is demonstrated in figure 3c, where the PED increases from 1.47 to 1.7 after rewiring, while in figure 3a, the AED increases from 15.4 to 15.5.

For the addition principle, one might expect these measures to satisfy it, since increasing node degrees consistently should preserve expected differences. The measures indeed respond differently: $H_{\text{PED}}$ satisfies the principle because its independence assumption ($P(k_{i},l_{j}|i,j\in E)=P(k_{i})P(l_{j})$) ensures that uniform degree increases preserve both distribution and absolute differences (proof in appendix C). By contrast, $H_{\text{AED}}$ can increase after uniform degree addition because new edges form, contributing to the expected difference, even if having a small contribution because of the conditional degree distribution $P(k_{i},l_{j}|i,j\in E)$ or absolute degree difference to their neighbouring nodes. This sensitivity to neighbourhood changes mirrors its behaviour under the transfer principle.

Under the replication principle, expected difference measures follow patterns identical to those of (adjacent) pairwise dispersion aggregation measures. Rather than remaining unchanged, they scale proportionally with network replication ($H_{\text{AED}}$ scales linearly with the replication factor while $H_{\text{PED}}$ scales quadratically). This scaling occurs because replication creates multiple disconnected components, which these measures interpret as increased heterogeneity—a property that could be valuable in applications requiring sensitivity to network fragmentation.

*Special heterogeneity* measures exhibit distinctive behaviours across the three network principles, reflecting their unique mathematical foundations.

The degree heterogeneity measure $H_{\text{J}}$ (cf. equation (4.28)) quantifies degree diversity by assessing frequency distribution of degrees. Under the transfer principle, $H_{\text{J}}$ can breach expectations when rewiring creates a more evenly distributed degree pattern. In figure 3b, heterogeneity increases from 0.38 to 0.41 after rewiring, as the degree distribution becomes more uniform, changing from (0.00, 0.55, 0.30, 0.00, 0.10, 0.05) to (0.00, 0.55, 0.25, 0.05, 0.15). Notably, $H_{\text{J}}$ remains unchanged when rewiring produces a permutation of the original degree distribution, indicating its insensitivity to structural changes that preserve degree frequency.

By contrast, $H_{\text{J}}$ fully complies with the addition principle because adding the same constant to all node degrees preserves the shape of the degree distribution. This demonstrates that as long as the degree distribution structure remains constant, changes in connectivity patterns or total edge count do not affect this measure’s evaluation. $H_{\text{J}}$ also complies fully with the replication principle because it depends solely on degree distribution, which remains unchanged during replication.

The graph heterogeneity measure $H_{\text{CS}}$ (cf. equation (4.27)) quantifies heterogeneity through deviation from regularity, measuring the difference between the adjacency matrix’s largest eigenvalue and mean degree. Under the transfer principle, $H_{\text{CS}}$ generally does not comply because rewiring does not necessarily transform the graph towards regularity. Figure 3c shows heterogeneity increasing from 1.47 to 1.49 after rewiring. However, when rewiring occurs from a more influential node to a less influential node (by eigenvector centrality), this measure decreases (proof in appendix C), capturing aspects beyond simple degree changes.

For the addition principle, $H_{\text{CS}}$ shows inconsistent behaviour because although uniform degree increase raises the mean degree accordingly, the addition principle does not specify how new edges connect nodes. Because multiple network topologies can exist for a given degree distribution, each potentially yielding different eigenvalues, $H_{\text{CS}}$ could increase, decrease, or remain unchanged depending on the specific topology. $H_{\text{CS}}$ breaches the replication principle by design, as it cannot be applied to the disconnected structures created through replication, revealing a fundamental limitation of this spectral-based heterogeneity measures.

These contrasting behaviours highlight the importance of understanding measure-specific mathematical properties when selecting appropriate heterogeneity measures for network analysis.

## 6. Conclusion

This study advances the systematic analysis of network heterogeneity through three foundational steps: synthesizing existing definitions into a unified conceptual framework, classifying measures into mathematically coherent classes, and evaluating these classes against desirable and necessary properties. The resulting framework resolves ambiguities in heterogeneity measurement while providing actionable guidance for researchers.

A key contribution lies in formalizing three perspectives on heterogeneity through conceptual synthesis. By analysing existing definitions, I distinguish degree heterogeneity (variability in node connectivity), topological heterogeneity (structural irregularities in edge configurations) and centrality and position-based heterogeneity (variations in nodes’ positional influence and structural roles within the network). The inclusion of a broader concept of heterogeneity provides important insights about the conceptual differences.

Mathematically, I derive six measure classes: global dispersion aggregation, (adjacent) pairwise dispersion, (adjacent) PED and special measures, by identifying unifying formulas under which existing measures fall through specific function choices. This classification reveals previously unrecognized relationships between supposedly distinct measures, such as the degree variance and Freeman centrality.

Systematic evaluation against properties (table 5) clarifies critical trade-offs. While most measures differentiate ER and PL networks, sensitivity depends on parameters like mean degree and the degree exponent γ. Only $H_{\text{S}}$ satisfies all network manipulation principles, whereas $H_{\text{J}}$ uniquely inverts star/diverse rankings, which is a finding that underscores how mathematical formulations and different conceptual approaches implicitly prioritize different aspects of heterogeneity.

**Table 5.** Cross-evaluation overview of analysis.

measure	measure type	graph model analysis	regular graph behaviour	diverse-star differentiation	principle alignment
**global dispersion aggregation**					
$H_{\text{S}}$	degree	differentiates ER/PL networks; weak for γ≥3	zero when degree equals mean	moderate distinction	complies with all three principles
$H_{\text{N}}$	degree	differentiates ER/PL networks; weak for low mean degrees	zero when all degrees equal mean	moderate distinction	addition only
$H_{\text{F}}$	degree	strong differentiation of ER/PL; weak for γ≥3	zero when all degrees identical	strong contrast; identifies stars as most heterogeneous	addition only
**adjacent pairwise dispersion**					
$H_{\text{A}}$	graph	differentiates ER/PL networks; weak for low mean degrees and when γ≥3	zero through identity of adjacent degree pairs	moderate distinction	addition only
$H_{\text{E}}$	graph	differentiates ER/PL networks; stable across changing mean degrees; weak for γ≥3	zero through identity of adjacent degree pairs	strong topology contrast	addition only
**pairwise dispersion**					
$H_{{\text{A}}_{\text{p}}}$	degree	differentiates ER/PL; weak for low mean degrees	zero when all nodes have equal degrees	inconsistent differentiation	addition, transfer
$H_{{\text{E}}_{\text{p}}}$	degree	differentiates ER/PL; weak for γ≥3	zero when all nodes have equal degrees	inconsistent differentiation	addition only
**expected difference**					
$H_{\text{AED}}$	graph	differentiates ER/PL networks; weak for γ≥3	zero from equal adjacent degrees	strong topology contrast	one
$H_{\text{PED}}$	degree	differentiates ER/PL; weak for low mean degrees	zero when degree distribution uniform	inconsistent differentiation	addition only
**special measures**					
$H_{\text{J}}$	degree	inconsistent differentiation; often reverses expected rankings	zero through uniform degree distribution	strong topology contrast; identifies stars as homogeneous	addition, replication
$H_{\text{CS}}$	graph	differentiates ER/PL networks; weak for γ≥3	zero when graph is regular (spectral property)	moderate distinction	none

Based on these findings, researchers should select heterogeneity measures according to their analytical focus and research objectives. For *degree heterogeneity*, global dispersion measures ($H_{\text{N}}$, $H_{\text{S}}$) effectively capture variation around mean degree for applications like epidemic modelling, while pairwise measures ($H_{\text{A}\text{p}}$, $H_{\text{E}\text{p}}$, $H_{\text{PED}}$) suit global assessments where differences in all potential connections matter. The special measure $H_{\text{J}}$ uniquely quantifies degree variety, suitable for examining structural complexities in dynamical systems. For *topological heterogeneity*, adjacent pairwise measures ($H_{\text{A}}$, $H_{\text{E}}$) can be useful in infrastructure analysis where neighbouring connections matter, while the AED measure $H_{\text{AED}}$ could also provide valuable insights for social network community formation. The special measure $H_{\text{CS}}$ effectively captures local topological information through network spectra. *Centrality-based* questions are best addressed with global dispersion measures like $H_{\text{F}}$ or $H_{\text{HG}}$, which quantify central node dominance and prove valuable for identifying influential individuals in social networks.

Two key limitations should be acknowledged. First, evaluations used controlled network models; real-world systems with community structure or temporal dynamics may exhibit different behaviours. Second, the diverse-star comparison focused on small networks, leaving open questions about scaling effects. Future work should develop hybrid measures combining degree and structural variability, test framework scalability on large real-world networks and expand principle compliance analysis to dynamic systems.

By bridging definitional clarity with operational guidance, this work establishes a foundation for rigorous, context-aware heterogeneity analysis. It demonstrates how the choice of measure critically shapes structural interpretation, whether in tracking epidemic spread through degree variability, optimizing infrastructure using topological aspects, or mapping power structures with centrality measures. Together, these contributions advance the methodology of network science.

## Figures and Tables

**Figure 4.**
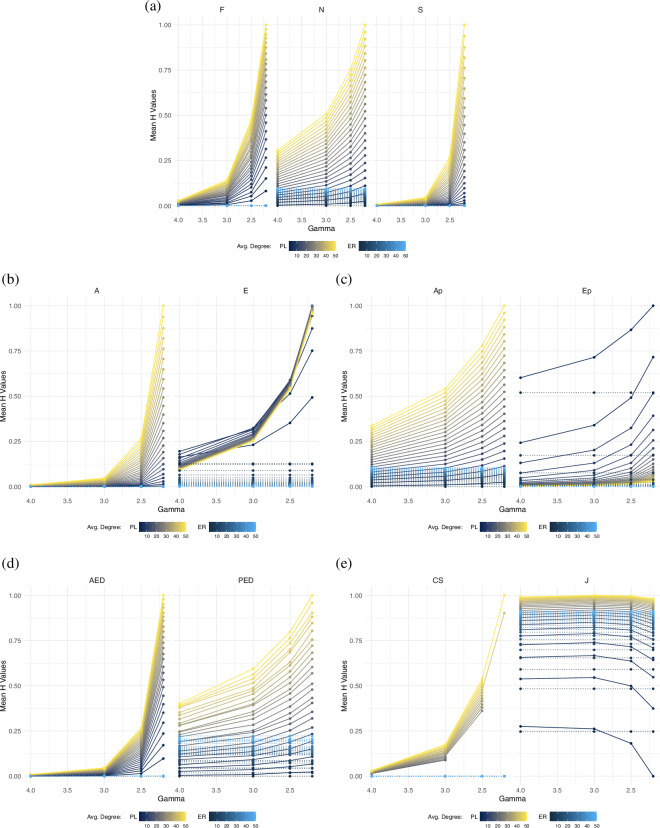
The plots show the evaluation of the simulated ER and PL networks for decreasing degree exponent values gamma and mean degree ranging from 2 to 50. Each plot shows measures scaled to the unit interval based on the minimum and maximum values across all networks for each class: (a) global dispersion aggregation measures $H_{\text{F}}$, $H_{\text{N}}$ and $H_{\text{S}}$; (b) adj. pairwise dispersion aggregation measures $H_{\text{A}}$ and $H_{\text{E}}$; (c) pairwise dispersion aggregation measures $H_{{\text{A}}_{\text{p}}}$ and $H_{{\text{E}}_{\text{p}}}$; (d) expected difference measures $H_{\text{AED}}$ and $H_{\text{PED}}$; and (e) special measures $H_{\text{CS}}$ and $H_{\text{J}}$. All, but the Jacobs measure fulfil the claim to assign higher heterogeneity values on average for PL networks than ER networks and in particular as the degree exponent decreases.

**Figure 6.**
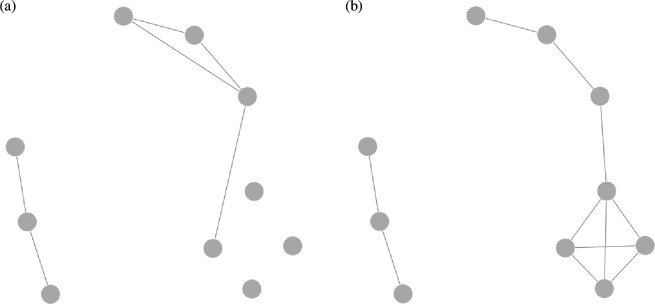
Adding one outgoing edge to each node of the network is expected to not increase the heterogeneity in the network. However, I find a counter-example in which this is the case for the adjacent expected difference. One reason for this could be that the measure not only takes the degrees, but also the network topology into account by measuring the expected difference for neighbouring nodes only. (a) Network before adding node degrees. The network has the degree sequence (3, 2, 2, 2, 1, 1, 1, 0, 0, 0) and heterogeneity value *H*_AED_(*G*) = 1.25, where AED is calculated using the uniform distribution for the degrees. (b) New network with increased node degrees. The network has the degree sequence (4, 3, 3, 3, 2, 2, 2, 1, 1, 1) and heterogeneity value *H*_AED_(*G*) = 1.6, where AED is calculated using the uniform distribution for the degrees.

## Data Availability

This article has no additional data.

## Footnotes

^1^
An overview of the results for all measures is presented in appendix A in figure 4.

## Appendix A. Simulation results

See figures 4 and 5.

## Appendix B. Regular graph zero-value conditions and proof

### B.1. Regular graph zero-value conditions

See table 6.

**Table 6.** Conditions and complying measures for zero heterogeneity in regular graphs. (Note: *r* = node degree, *h*(*K*) = network statistic, *c* = scaling factor, *g* = convex function.)

measure class	conditions	measures
global dispersion aggregation	r−h(K) equals a root of g	$H_{\text{F}}$, $H_{\text{N}}$, $H_{\text{S}}$
(adjacent) pairwise dispersion aggregation	c>0	$H_{\text{A}}$, $H_{\text{E}}$, $H_{{\text{A}}_{\text{p}}}$, $H_{{\text{E}}_{\text{p}}}$
expected difference	c>0	$H_{\text{AED}}$, $H_{\text{PED}}$
special heterogeneity measures	—	$H_{\text{CS}}$, $H_{\text{J}}$

### B.2. Proposition and proof for regular graph homogeneity

**Proposition**: let $G_{r}$ be an r-regular graph with degree r and let h(K) be the network statistic. For global dispersion aggregation measures with convex function g introduced in equation (4.1):

(i) if g has a unique root at $x^{\prime }$, then the heterogeneity measure equals 0 if and only if $r-h(K)=x^{\prime }$; and
(ii) if g has multiple roots $\left\{x_{1}^{\prime },x_{2}^{\prime },\ldots \;\right\}$*,* then the heterogeneity measure equals 0 if and only if r−h(K) equals one of these roots.

This proposition shows that regular graphs are classified as completely homogeneous (measure = 0) only under specific conditions relating to the network’s degree r, the statistic h(K), and the roots of the convex function g. For the measure to equal zero, the difference between the degree and the statistic needs to equal the function’s roots. *Proof*: let $G_{r}$ be an r-regular graph. Then it holds:

(B 1)H(Gr)=c∑i=1ng(ki−h(K)),(B 2)=c∑i=1ng(r−h(K)),(B 3)=cng(r−h(K)).

c, and n are positive values, therefore:

(B 4)
$$\begin{array}{lcr} & H(G)\overset{\text{!}}{=}0\Leftrightarrow g(r-h(K))=0. & \end{array}$$

## Appendix C. Counter-examples and proofs of the principles

### C.1. Sufficient conditions for principle compliance

See table 7.

**Table 7.** Sufficient conditions under which measures in the dispersion aggregation class satisfy the transfer, addition and replication principles.

principle	dispersion aggregation	(adjacent) pairwise
	global	
**transfer**	$h(K)=h(K^{\prime })=:s\wedge \left(\sum_{i=m,l}g(k_{i}-s)-g(k_{i}^{\prime }-s)>0\;\;\vee \;\;g{|}_{R_{\geq 0}}\right)$	f identity, g absolute
**addition**	$h(K^{\prime })=h(K)+\Delta k\;\;\vee \;\;\left(h(K^{\prime })\leq h(K)\wedge g↗\right)$	f identity function
**replication**	$h(K)=h(K^{\prime })$ and $x=c/c^{\prime }$	—

### C.2. Transfer principle

Let {i,m}∈E and {i,l}∉E, where $k_{m}>k_{l}+1$. Rewiring from node m to l leads to the new network $G^{\prime }=(V,E\cup \{i,l\}\setminus \{i,m\})=\left(V,E^{\prime }\right)$, where $k_{m}^{\prime }=k_{m}-1,k_{l}^{\prime }=k_{l}+1$, and therefore $k_{m}^{\prime }\geq k_{l}^{\prime }$.

#### C.2.1. Dispersion aggregation

*Global dispersion aggregation.* The behaviour of the measure after rewiring depends heavily on the shape of the convex function g and the statistic h. In particular, because of the relational aspect the transfer principle is easily breached.

In the following special cases, the behaviour of the measure owing to rewiring is as expected, that is, assigning a lower value to the network after rewiring.

Under the assumption that the statistic’s value does not change and for the convex function holds $g(k_{m}-h(K))+g(k_{l}-h(K))>g(k_{m}^{\prime }-h(K))+g(k_{l}^{\prime }-h(K))$, the heterogeneity measure does decrease:

(C 1)H(G)=∑i=1ng(ki−h(K)),(C 2)=∑i:i≠m,lg(ki−h(K))+g(km−h(K))+g(kl−h(K)),(C 3)>∑i:i≠m,lg(ki−h(K′))+g(km′−h(K′))+g(kl′−h(K′)),(C 4)=∑i=1ng(ki′−h(K′)),(C 5)=H(G′).

This scenario is the case for example for functions g that are restricted to ${\mathbb{R}}_{\geq 0}$ and $h(K)=h(K^{\prime })$.

*Statistic of interest: maximum degree*

Case 1: $k_{m}<k_{\text{max}},$

(C 6)HHG(G)=∑i=1n(kmax−ki),(C 7)=∑i:i≠m,l(kmax−ki)+kmax−km+kmax−kl,(C 8)=∑i:i≠m,l(kmax−ki)+kmax−(km−1)+kmax−(kl+1),(C 9)=∑i=1n(kmax−ki′)(C 10)=HHG(G′).

This case shows that this measure is not affected by the rewiring process and therefore is not affected by other high degree nodes—as long as those have a lower degree than the maximum degree.

Case 2: $k_{m}=k_{\text{max}},$

(C 11)HHG(G)=∑i=1n(kmax−ki),(C 12)=nkmax−∑i=1nki,(C 13)=nkmax−(∑i:i≠m,lki+km−1+kl+1),(C 14)=nkmax−∑i=1nki′,(C 15)>n(kmax−1)−∑i=1nki′,(C 16)=nkmax′−∑i=1nki′,(C 17)=∑i=1n(kmax′−k′),(C 18)=HHG(G′).

Therefore, the transfer principle is fulfilled in case of rewiring an edge that is incident to the node with highest degree in the network.

*Statistic of interest: mean degree*

(C 19)H(G)=∑i=1ng(ki−k¯)(C 20)=∑i=1ng(ki−n−1∑jkj)(C 21)=∑i:i≠m,lg(ki−n−1(∑j:i≠m,lkj+km+kl))+g(km−k¯)+g(kl−k¯)>∑i:i≠m,lg(ki−n−1(∑j:i≠m,lkj+km−1+kl+1))(C 22)+g(km−1−k¯)+g(kl+1−k¯)(C 23)=∑i=1ng(ki′−k¯′)(C 24)=H(G′).

The transfer principle is only fulfilled, if

(C 25)
$$\begin{array}{lcr} & g(k_{m}-\bar{k})+g(k_{l}-\bar{k})>g(k_{m}-1-\bar{k})+g(k_{l}+1-\bar{k}). & \end{array}$$

Case: pairwise dispersion aggregation

The Albertson measure, adapted to summing over all pairs of nodes, fulfils the transfer principle—that is, it decreases:

(C 26)HAp(G)=∑i,j∈V|ki−kj|(C 27)=∑i,j∈V∖{m,l}|ki−kj|+2∑i∈V∖{m,l}|ki−km|+2∑i∈V∖{m,l}|ki−kl|+2|km−kl|(C 28)=∑i,j∈V∖{m,l}|ki′−kj′|+2∑i∈V∖{m,l}: ki≤kl|ki−km|+2∑i∈V∖{m,l}: kl<ki<km|ki−km|+2∑i∈V∖{m,l}: ki≥km|ki−km|+2∑i∈V∖{m,l}: ki≤kl|ki−kl|+2∑i∈V∖{m,l}: kl<ki<km|ki−kl|+2∑i∈V∖{m,l}: ki≥km|ki−kl|+2|km−kl|

(C 29)=∑i,j∈V∖{m,l}|ki′−kj′|+2∑i∈V∖{m,l}: ki≤kl(|ki′−km′|+|ki′−kl′|)+2∑i∈V∖{m,l}: kl<ki<km(|ki′−km′|+|ki′−kl′|+2)+2∑i∈V∖{m,l}: ki≥km(|ki′−km′|+|ki′−kl′|)+2|km′−kl′|+4(C 30)=HAp(G′)+4|{i∈V:kl<ki<km}|+4(C 31)>HAp(G′).

The adapted Estrada measure does not always behave under transfer as expected. In case of chosen parameter c=1, functions $g(\cdot )=\cdot^{2}$ and $f(\cdot )=\cdot^{-0.5}$, the measure increases after rewiring. For the networks in figure 3b the measure yield $H_{{\text{E}}_{\text{p}}}(G)=15.4$ and $H_{{\text{E}}_{\text{p}}}(G^{\prime })=15.7$.

#### C.2.2. Special heterogeneity measures

The measure of [10] does not decrease after rewiring an edge from a higher degree node to a lower degree node. The network shown in figure 3b is a counter-example, in which the graph has a value of 0.38 and increases through the transfer process to a value of 0.41. In general, the measure remains unchanged if the degree distribution after rewiring is a permutation of the initial one.

The measure of [13] does not increase because of the rewiring.

Let A be the adjacency matrix of the given graph G and $A^{\prime }$ the adjacency matrix of the graph $G^{\prime }$ resulting from rewiring the edge {i,m} to {i,l}. Both are real and symmetric and therefore Hermitian matrices. Their spectra are ${ℵ}_{1}\geq \ldots \geq {ℵ}_{n}$ and ${ℵ}_{1}^{\prime }\geq \ldots \geq {ℵ}_{n}^{\prime }$ with ${ℵ}_{i},{ℵ}_{i}^{\prime }\in \mathbb{R}$ and $x\in {\mathbb{R}}^{n}$ the dominant eigenvector of A. The transfer can be represented by a perturbation matrix E, defined with entries $e_{ij}=e_{li}=1$, $e_{im}=e_{mi}=-1$ and $e_{ij}=0$,otherwise. Then it is $A^{\prime }=A+E$.

With the Rayleigh quotient I can derive:

(C 32)ℵ1′=max‖y‖=1y⊤A′y,(C 33)≥x⊤A′x,(C 34)=x⊤Ax+x⊤Ex,(C 35)=ℵ1−2xi(xm−xl).

The Perron–Frobenius theorem states that for a real, irreducible, non-negative square matrix it exists one eigenvector $x\in {\mathbb{R}}^{n}$ of matrix A with only positive entries and that this is the dominant eigenvector [36,37].

Additionally, the entries of the dominant eigenvector represent the importance and influence of the corresponding node in the network (eigenvector centrality). The importance is not only captured by the degree, but also by the neighbouring nodes and a higher value can be assigned to nodes with few, but high degree neighbouring nodes or for high-degree nodes with neighbours that not necessarily have high degrees [38]. Under the assumption that the effect of the neighbours of nodes m and l is little, I can conclude, that $x_{l}\leq x_{m}$ and therefore the dominant eigenvalue decreases because of the transfer.

Altogether it follows that the heterogeneity measure decreases in case of rewiring the edge from a more influential node to a less influential node in that sense and therefore fulfils the transfer principle:

(C 36)HCS(G)=ℵ1−k¯,(C 37)=ℵ1−k′¯,(C 38)≥ℵ1′−k′¯,(C 39)=HCS(G′).

However, the transfer principle as stated by [7] is not fulfiled by the measure as shown in figure 3c. A rewiring of an edge from a node with high degree that is not influential, to a node with low degree, but is influential, can increase the measure’s value. This example shows that it is important to determine meaningful heterogeneity and its relationship to the measure’s behaviour.

### C.3. Addition principle

The addition principle considers the situation in which all node degrees are increased by the same amount, that is $k_{i}^{\prime }=k_{i}+1,\forall i\in V$, and the heterogeneity measure is expected to not change [7].

#### C.3.1. Dispersion aggregation

*Global dispersion aggregation.* Depending on the statistic function h and the actual progression of the convex function g, the value of the general heterogeneity measure vary and the addition principle does not hold. In the special case, where $h(K^{\prime })=h(K)+1$ holds, the measure does not change. This is the case for example for statistics like the mean, maximum and minimum degree. Assuming c=1:

(C 40)H(G)=∑i=1ng(ki−h(K)),(C 41)=∑i=1ng(ki+1−1+h(K)),(C 42)=∑i=1ng((ki+1)−(h(K)+1)),(C 43)=∑i=1ng(ki′−h(K′)),(C 44)=H(G′).

Another exception is, if g is a monotone function and $h(K)⪋h(K^{\prime })$. Without loss of generality, assume that g is monotone increasing and $h(K)\leq h(K^{\prime })$. Then:

(C 45)H(G)=∑i=1ng(ki−h(K)),(C 46)≥∑i=1ng(ki−h(K′)),(C 47)≥∑i=1ng(ki+1−h(K′)),(C 48)=∑i=1ng(ki′−h(K′)),(C 49)=H(G′).

*Statistic of interest: maximum degree*

(C 50)HHG(G)=∑i=1n(kmax−ki),(C 51)=∑i=1n(kmax+1−1−ki),(C 52)=∑i=1n((kmax+1)−(ki+1)),(C 53)=∑i=1n(kmax′−ki′),(C 54)=HHG(G′).

*Statistic of interest: mean degree*

(C 55)HHG(G)=∑i=1n(ki−k¯)(C 56)=∑i=1n(ki−n−1∑jkj)(C 57)=∑i=1n(ki+1−1−n−1∑jkj)(C 58)=∑i=1n((ki+1)−(n−1∑jkj+1))(C 59)=∑i=1n(ki′−k¯′)(C 60)=HHG(G′)

(*Adjacent*) *pairwise dispersion aggregation.* The heterogeneity measure does change much and in case of function f being the identity function it does not change at all:

(C 61)H(G)=c∑{i,j}∈Eg(ki−kj),(C 62)=c∑{i,j}∈Eg(ki+1−1−kj),(C 63)=c∑{i,j}∈Eg((ki+1)−(kj+1)),(C 64)=c∑{i,j}∈Eg(ki′−kj′),(C 65)=H(G′).

#### C.3.2. Expected difference

*Adjacent expected difference.* The measure can increase as shown in the counter-example in figure 6.

*Pairwise expected difference.* It is easy to see that the heterogeneity measure does not change because of the addition transfer:

(C 66)HPED(G)=c∑i,j∈V|ki−lj|P(ki)P(lj),(C 67)=c∑i,j∈V|ki+1−1−lj|P(ki)P(lj),(C 68)=c∑i,j∈V|(ki+1)−(lj+1)|P(ki)P(lj),(C 69)=c∑i,j∈V|(ki+1)−(lj+1)|P(ki+1)P(lj+1),(C 70)=c∑i,j∈V|ki′−lj′|P(ki′)P(lj′),(C 71)=HPED(G′).

#### C.3.3. Special heterogeneity measures

The transformation of the addition principle does not change the value of the Jacob measure, which can be easily seen:

(C 72)HJ(G)2=1n∑kminkmax(1−P(k))2, P(k)≠0,(C 73)=1n∑kmin+1kmax+1(1−P′(k))2, P′(k)≠0,(C 74)=HJ(G′)2.

The measure of Collatz and Sinogowitz is based on the calculation of the dominant eigenvalue and the mean degree. The addition principle increases the mean degree by one, but because it does not specify how the added edges are connected within the network, I cannot conclude how the dominant eigenvector, which reflects the importance and influence of the nodes and their neighbours, changes.

### C.4. Replication principle

#### C.4.1. Dispersion aggregation

*Global dispersion aggregation.* Let G=(V,E) be a network with n nodes. Without loss of generality suppose $G^{\prime }=\left(V^{\prime },E^{\prime }\right)$ is an integer-multiple by factor x of network G. If $h(K^{\prime })=h(K)$ and $c^{\prime }=x^{-1}c$, it holds:

(C 75)H(G′)=c′∑i=1n′g(ki′−h(K′)),(C 76)=x−1c∑i=1xng(ki′−h(K′)),(C 77)=x−1c∑i=1nxg(ki−h(K)),(C 78)=H(G).

If $c^{\prime }$ does not equal $x^{-1}c$, the value of the measure is simply a multiple of the initial value. This is, for example the case for the Høivik and Gleditsch measure (cf. equation (4.2)) and the Nikiforov measure (cf. equation (4.12)).

The Freeman measure (cf. equation (4.5)) breaches the replication principle because of the normalization step with the smallest total difference for a graph with n points. The problem here is that their scaling constant depends on the number of nodes. The value decreases:

(C 79)HF(G′)=1c′∑i=1n′(kmax′−ki′),=1[(n+x)−2][(n+x)−1]x(C 80)×∑i=1n(kmax−ki),=x(n−2)(n−1)+x[(n−2)(n−1)]+x2(C 81)×∑i=1n(kmax−ki),=1(n−2)(n−1)x+(n−2)(n−1)+x(C 82)×∑i=1n(kmax−ki),(C 83)≥1(n−2)(n−1)∑i=1n(kmax−ki),(C 84)=HF(G).

## Appendix D. Overview of network statistics

See table 8.

**Table 8.** Network statistics and degree sequences for the diverse and star graphs.

graph type	label	no. nodes (n)	no. edges (m)	edge density ($\frac{2m}{n(n-1)}$)	degree sequence
diverse	(4, 4)	4	4	0.67	1, 2, 2, 3
diverse	(5, 4)	5	4	0.40	0, 1, 2, 2, 3
diverse	(5, 6)	5	6	0.60	1, 2, 2, 3, 4
diverse	(7, 12)	7	12	0.57	1, 2, 3, 3, 4, 5, 6
star	(4)	4	3	0.50	1, 1, 1, 3
star	(5)	5	4	0.40	1, 1, 1, 1, 5
star	(6)	6	5	0.33	1, 1, 1, 1, 1, 5
star	(7)	7	6	0.29	1, 1, 1, 1, 1, 1, 6
